# Metallization of Targeted Protein Assemblies in Cell‐Derived Extracellular Matrix by Antibody‐Guided Biotemplating

**DOI:** 10.1002/advs.202302830

**Published:** 2023-10-18

**Authors:** Chang Woo Song, Jaewan Ahn, Insung Yong, Nakhyun Kim, Chan E Park, Sein Kim, Sung‐Yoon Chung, Pilnam Kim, Il‐Doo Kim, Jae‐Byum Chang

**Affiliations:** ^1^ Department of Materials Science and Engineering Korea Advanced Institute of Science and Technology (KAIST) 291 Daehak‐ro Daejeon 34141 Republic of Korea; ^2^ Department of Bio and Brain Engineering Korea Advanced Institute of Science and Technology (KAIST) 291 Daehak‐ro Daejeon 34141 Republic of Korea; ^3^ Department of Biomedical Engineering Sungkyunkwan University (SKKU) Suwon 16419 Republic of Korea; ^4^ Department of Biological Sciences Korea Advanced Institute of Science and Technology (KAIST) 291 Daehak‐ro Daejeon 34141 Republic of Korea

**Keywords:** antibody, biotemplating, extracellular matrix, hydrogen, metallization

## Abstract

Biological systems are composed of hierarchical structures made of a large number of proteins. These structures are highly sophisticated and challenging to replicate using artificial synthesis methods. To exploit these structures in materials science, biotemplating is used to achieve biocomposites that accurately mimic biological structures and impart functionality of inorganic materials, including electrical conductivity. However, the biological scaffolds used in previous studies are limited to stereotypical and simple morphologies with little synthetic diversity because of a lack of control over their morphologies. This study proposes that the specific protein assemblies within the cell‐derived extracellular matrix (ECM), whose morphological features are widely tailorable, can be employed as versatile biotemplates. In a typical procedure, a fibrillar assembly of fibronectin—a constituent protein of the ECM—is metalized through an antibody‐guided biotemplating approach. Specifically, the antibody‐bearing nanogold is attached to the fibronectin through antibody–antigen interactions, and then metals are grown on the nanogold acting as a seed. The biomimetic structure can be adapted for hydrogen production and sensing after improving its electrical conductivity through thermal sintering or additional metal growth. This study demonstrates that cell‐derived ECM can be an attractive option for addressing the diversity limitation of a conventional biotemplate.

## Graphical abstract

In this study, a thin metallic film, which imitates the fibrillar assembly of fibronectin, a type of cell‐derived extracellular matrix (ECM) protein, is synthesized using antibody‐guided biotemplating. The resulting metalized ECMs, along with the fibronectin assemblies, are rendered electrically conductive through thermal sintering or additional metal growth, and then are applicable to hydrogen production and sensing.

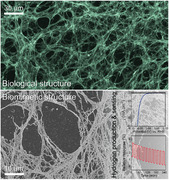

## 1 Introduction

Biological systems possess highly hierarchical 3D structures made up of a large number of different proteins. These systems exhibit significantly greater levels of structural complexity than man‐made materials, leading to unique properties.^[^ ^1^, ^2^ ^]^ To exploit this complexity for synthesizing functional materials, researchers have developed a technique called biotemplating.^[^ ^3^ ^]^ Biotemplating involves using a part^[^ ^4^ ^]^ or all of a given biological system,^[^ ^5^, ^6^, ^7^, ^8^, ^9^, ^10^, ^11^ ^]^ as well as biological systems that have been patterned or assembled,^[^ ^12^, ^13^ ^]^ as templates to synthesize materials. Numerous studies have reported the fabrication of biocomposites using biotemplating by combining the mechanical, electrical, and catalytic functionalities of inorganic materials with the structural advantages of biotemplate.^[^ ^14^ ^]^ Of these, the creation of electrically conductive biocomposites has garnered significant interest. To impart the electrical conductivity to insulative biological scaffolds, electroless plating or physical vapor deposition of metal onto them, such as on viruses,^[^ ^15^, ^16^ ^]^ aggregated algae,^[^ ^17^, ^18^ ^]^ extracted plant vessels,^[^ ^19^, ^20^ ^]^ or single mammalian cells,^[^ ^9^ ^]^ as well as the in vitro assembly of proteins,^[^ ^21^, ^22^, ^23^ ^]^ have been successful in synthesizing a variety of electrically conductive biomimetic materials. However, these templates are limited in their ability to realize diverse metallic structures because of the lack of a means to control their porosity, alignment, or 3D organization.

To overcome this limitation, we aimed to explore the use of a cell‐derived extracellular matrix (ECM) as a template to create electrically conductive architecture that can be customized to meet user demands. The ECM is a non‐cellular biological scaffold found within tissues playing a critical role in cell survival by providing mechanical support, facilitating cell migration, and transducing mechanical signals.^[^ ^24^, ^25^, ^26^ ^]^ It is comprised of various fibrillar and non‐fibrillar proteins, such as collagen, elastin, and fibronectin, all of which are organized into a fibrous network. In recent years, numerous studies have reported methods for controlling the chemical and physical properties of ECM, such as porosities, molecular contents, alignments, and modulus, as well as for using 3D printing to create ECM that more closely resemble the native ECM or provide a customized environment for the cells.^[^ ^27^, ^28^, ^29^, ^30^, ^31^ ^]^ Such engineered ECMs have been widely used in the development of artificial organs, in vitro modeling of diseases, and pharmaceutical screening systems to replace animal testing. In addition, the use of various cell types, including muscle, endothelial, and neural systems, among others, or different culture conditions can lead to the creation of versatile ECM configurations.^[^ ^32^ ^]^ Given its highly diverse structures and controllability, cell‐derived ECM is an attractive option for biotemplating. However, to date, there have not been any reported attempts to utilize them as scaffolds for synthesizing electrically conductive materials.

Recently, our group proposed the antibody‐guided biotemplating approach that relies on antibody–antigen interaction and electroless plating.^[^ ^33^ ^]^ Using our approach, we can demonstrate the synthesis of metal architectures that resemble specific protein assemblies within mammalian cells and tissues. In the present study, we further extend our biotemplating method to ECM derived from a fibroblast in an attempt to endow electrical conductivity into the metalized ECM along the fibrillar assembly of a selected protein called fibronectin. Notably, fibronectin was selected as the biotemplate among the various proteins that compose the ECM because it is abundantly formed as a connected fibrous structure within the fibroblast ECM.^[^ ^34^ ^]^ Selective protein metallization within the ECM allows us to choose the appropriate protein assembly as a template to fabricate an electrically conductive biomimetic film. To metalize the fibronectin specifically, we initially carried out decellularization, which detaches the cell while retaining its ECM. The decellularized ECM (dECM) was stained with a fibronectin‐targeting primary antibody, and then a nanogold‐conjugated secondary antibody was attached to the primary antibody. Next, gold nanoparticles (AuNPs) were grown via electroless plating, wherein the nanogold served as seeds for a surface‐mediated reduction of the added Au.^[^ ^35^ ^]^ For imparting electrical conductivity, the dECMs metalized along with the fibronectin were post‐treated through thermal sintering, which induced a necking formation between the neighboring AuNPs, or by growing secondary metal phases (e.g., Pd or Pd‐Pt alloy) on the AuNP surface, which also provided additional functionalities. The fabricated electrically conductive dECM films were utilized as a platform for hydrogen evolution reaction (HER) and chemiresistive hydrogen gas sensing.

## 2 Results and Discussion

### 2.1 Synthesis and Characterization of Metalized dECM by Antibody‐Guided Biotemplating

Among the different kinds of proteins in the ECM, we selected fibronectin as a scaffold secreted by an NIH/3T3 cell, a type of fibroblast known to produce abundant ECM components in connective tissue.^[^ ^36^ ^]^ Fibronectin usually exists as a soluble dimeric biomolecule in bodily fluids. Integrins on the cell surface can induce end‐to‐end linear assembly of the fibronectin dimers, resulting in the formation of an insoluble fibrillar structure.^[^ ^37^, ^38^ ^]^ Fibrillar fibronectin plays central roles in cellular biology, such as transducing mechanical signals to cells, regulating the distribution and proliferation of cells, and storing biomolecules.^[^ ^39^ ^]^ Such interconnected fibrillar fibronectin is sufficient to serve as the organized template for fabricating the metallic nano‐fibrous structure in this study.

**Figure 1:**
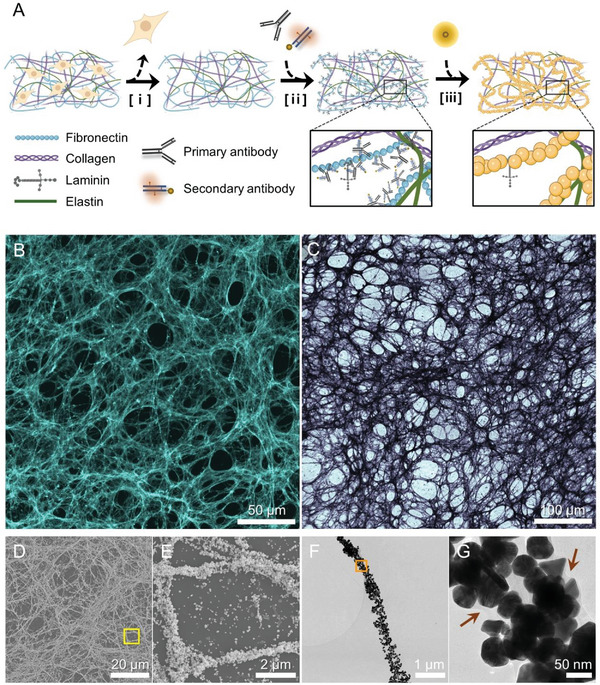
Schematic of the antibody‐guided biotemplating to metalize specific protein assemblies in dECM and the characterization of dECM after antibody staining and Au growth along with fibronectin. A) The procedures include i) decellularization and chemical fixation, ii) staining of the fibronectin by primary and secondary antibodies, and iii) Au growth process. B) Fluorescence image showing the structure of fibronectin after antibody staining. C) Bright‐field image of the metalized fibronectin structure after the Au growth step. D) SEM image of AuNP‐grown dECM. E) Enlargement of the yellow box in (D). F) TEM image of a single fiber in the metalized dECM. G) Enlargement of the orange box in (F). The brown arrows indicate the edges of the fiber, which consists of organic matter.

**Figure** **1**A depicts a procedural schematic of the synthesis of the biomimetic metallic structures taking on the fibronectin assemblies in the cell‐derived dECM. First, mouse embryonic fibroblasts (NIH/3T3 cells) were cultured into a full monolayer and treated with a surfactant mixture to detach the grown cells from their ECM.^[^ ^40^ ^]^ Scanning electron microscopy (SEM) was used to confirm that the reticulated configuration of the intact ECM, which is comprised of fibers with various diameters, was maintained even after decellularization (Figure S1, Supporting Information). Subsequently, the dECM was labeled with a primary antibody targeting the fibronectin, and then a secondary antibody bearing both fluorophores and nanogold (1.4 nm) was attached to the primary antibody. Finally, taking advantage of the catalyzing reduction effect on the nanogold surface, we specifically grew AuNPs on the nanogold that aligned with the fibronectin structures. AuNP growth using the catalyzing reduction effect on the metal surface was also possible on different metal surfaces, as shown in Figure S2 (Supporting Information), where AuNPs were grown using 3 nm diameter platinum nanoparticles (PtNPs) as seeds.

As shown in the fluorescence microscope image (Figure 1B), we can identify the densely intertwined fibrillar fibronectin structure with a microporous architecture obtained from NIH/3T3 cells. On the other hand, the other cell lines that were not fibroblast showed different fibronectin morphologies, with not sufficiently developed and not abundant enough (Figure S3, Supporting Information). Even after the growth of AuNPs, this distinct structural feature can be soundly preserved, as confirmed by the bright‐field optical microscope image (Figure 1C). Electron microscopy, including SEM, transmission electron microscopy (TEM), and scanning transmission electron microscopy (STEM), were used to further analyze the structure of the metalized dECM in the nanoscale. Figure 1D‒G shows that individual AuNPs decorated on fibronectin were arranged in a manner similar to the fibronectin identified in the fluorescence and bright‐field images. The high‐resolution TEM (HRTEM) image shows that the single fiber in the metalized dECM was ≈150 nm thick in diameter (Figure 1G). A region of weaker contrast that outlines the contour of the fiber structure, as indicated by the brown arrows in Figure 1G, can be ascribed to the organic component of the intrinsic ECM. In particular, with successive Au growth up to four iterations, we obtained gradually larger AuNPs from 30 nm (one iteration) to 50 nm (four iterations) in diameter (Figure S4, Supporting Information). Through additional Au growth, the metalized structure manifested a more definite fibrous architecture of the original fibronectin assembly while also decreasing the interparticle spacing between the AuNPs. In the end, the metalized dECM prepared by four iterative Au growth (Au‐ECM) was used in subsequent experiments, unless otherwise mentioned. Moreover, X‐ray diffraction (XRD) and energy dispersive X‐ray spectroscopy (EDS) analyses were carried out to ascertain the composition of the AuNPs grown on the dECM (Figure S5, Supporting Information).

We identified two essential factors for achieving the successful synthesis of densely packed fibrous AuNP arrays. The first is that antibody–antigen interaction allows for the selective metallization of specific protein architectures. With fibronectin as the reference, we explored the use of other types of ECM proteins as templates, such as those shown in Figure S6 (Supporting Information), finding that they display imprecise fibrous structures in the dECM or generate AuNPs with insufficient density. This result corroborates that fibronectin is the most suitable candidate for synthesizing metallic nano‐fibrous biocomposites. The second factor is that the labeling density of the AuNPs was improved through the in situ seed‐mediated growth of AuNPs on nanogold rather than through the direct labeling of proteins with antibodies bearing large colloidal AuNPs. To prove this, we prepared the dECM by staining with a primary antibody targeting fibronectin and a secondary antibody bearing 10 nm colloidal AuNPs, evaluating the AuNP labeling density. In Figure S7 (Supporting Information), the fluorescence image shows the analogous fibronectin structure seen in the previously identified fluorescence image (Figure 1B) in the case of using the secondary antibody‐bearing nanogold. However, a detailed observation with TEM showed that the actual labeling efficiency of the secondary antibodies substantially decreased in the case of secondary antibodies bearing 10 nm colloidal AuNPs compared with nanogold‐containing secondary antibodies. These results were consistent with previous reports indicating that antibody labeling efficiency decreased as the antibody was conjugated with large AuNPs.^[^ ^41^, ^42^ ^]^ Altogether, we can confirm that the labeling of specific proteins using antibodies and subsequent in situ growing AuNPs from the nanogold were both instrumental in obtaining dense AuNP‐arrays using dECM as a scaffold.

### 2.2 Imparting Electrical Conductivity to Au‐ECM and Its Functionalization for Utilization in HER

**Figure 2:**
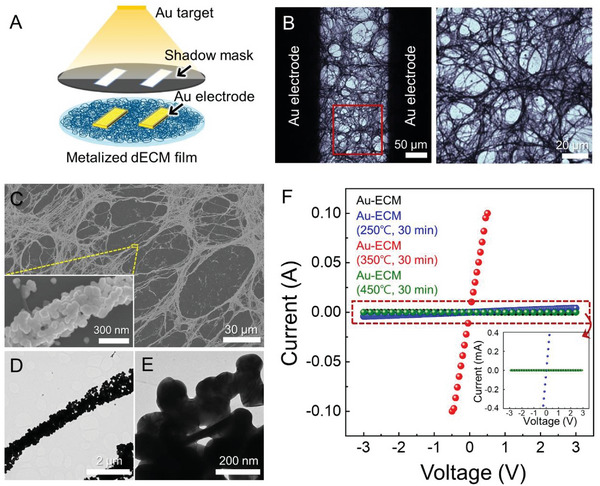
The endowment of electrical conductivity to Au‐ECM by thermal sintering. A) Schematic of the deposition of Au electrodes on top of the metalized dECM. B) Bright‐field image of the interface region between the Au electrodes (left panel) and enlargement of the red box (right panel). C) SEM image of Au‐ECM after thermal sintering at 350 °C; the inset is the magnification of the yellow box. D,E) TEM images of a single fiber in Au‐ECM treated with thermal sintering at 350°C. F) The current–voltage curves of Au‐ECMs are plotted as classified by the thermal sintering temperature.

We subsequently investigated the electrical properties of the Au‐ECM. As illustrated in **Figure** **2**A, we deposited a pair of rectangular‐shaped Au electrodes on top of the Au‐ECM, with each electrode separated by 200 µm and with a 4 mm width, to facilitate the conductivity measurements. The Au‐ECMs were sintered at 250, 350, and 450 °C for 30 min in air, respectively, prior to the electrical measurements. The thermal sintering was expected to induce necking between the AuNPs, increasing the inter‐particle contact area and their coalescence, which, in turn, would result in high electrical conductivity. Eventually, we determined the optimal sintering temperature condition as 350 °C, wherein the reticulated configuration can be maintained after thermal treatment, as identified in the bright‐field image in Figure 2B. The SEM image clearly showed the preserved Au‐ECM structure at the nanoscale, showing the slightly smoothed surface of the AuNPs (Figure 2C). In addition, the joints were identified between the neighboring AuNPs while bridging the sintered AuNPs and then making the fibrously connected AuNP arrays into nearly metallic rods that ensure an uninterrupted electron pathway (Figure 2D,E).

The electrical conductivity of the sintered Au‐ECM was determined from the current‐voltage (*I‒V*) response over a −3 to +3 V range using a two‐point probe approach. Notably, the conductance of sintered Au‐ECM substantially increased at the optimal condition (350 °C) by nearly 10^7^‐fold compared with Au‐ECM without sintering. These results indicate that the AuNP‐arrays in the Au‐ECM were insufficient to ensure the electron pathway across the 200 µm interval between the two Au electrodes; thus, the necking formation via thermal sintering was essential to fabricate conductive biotemplate films. In contrast, the conductivity of the Au‐ECM sintered at other temperatures (250 and 450 °C, respectively) did not show significant improvements. In fact, at 450 °C, the conductivity deteriorated compared with that of the non‐sintered Au‐ECM. Our analysis was corroborated by the SEM images (Figure S8, Supporting Information), which confirmed insufficient necking formation between the AuNPs for the samples sintered at 250 °C and the severely coalesced AuNPs for those sintered at 450 °C, both of which led to inadequate electrical pathways. In addition, the conductivity of the pristine dECM after sintering at 350 °C can hardly be measured, indicating that the underlying conductivity of the sintered Au‐ECM was derived from the interconnected AuNPs, not because of the combusted organic matter within the dECM (Figure S9, Supporting Information). We summarized the conductivity of Au‐ECM and sintered Au‐ECM (Table S1, Supporting Information) based on the surface profiler measurements of the thickness of the metalized dECM (Figure S10, Supporting Information).

**Figure 3:**
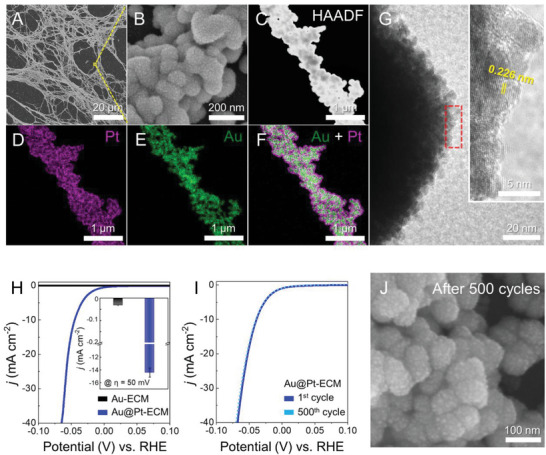
The synthesis of Au@Pt‐ECM and its electrocatalytic performance toward HER. A, B) SEM images of Au@Pt‐ECM. C‒F) HAADF image and EDS composition maps of Au@Pt‐ECM. G) HRTEM image of Au@Pt‐ECM at the edge region, presenting the tiny polycrystalline PtNPs. H) HER polarization curves plotting the current density normalized by the electrode area according to a potential change. The inset shows the measured current density of Au‐ECM and Au@Pt‐ECM at 50 mV overpotential. Data are presented as mean ±  SD, *n* = 5. I) Catalytic stability test of Au@Pt‐ECM over 500 repeated cycles. J) SEM image of Au@Pt‐ECM after 500 repeated cycles. All results were *iR*‐corrected.

We demonstrated that the sintered Au‐ECM could be further functionalized with PtNPs, which are referred to as Au@Pt‐ECM. To synthesize the PtNPs directly onto the sintered Au‐ECM (350 °C), we added an H_2_PtCl_6_·6H_2_O solution ionized to PtCl_6_^2−^ in deionized water (D.I.) to the sintered Au‐ECM. Subsequently, the L‐ascorbic acid solution was applied as a reductant. Due to the acidic conditions (pH ≈ 2.5) imposed by the L‐ascorbic acid, a positive charge formed around the Au‐ECM, attracting the negatively charged complex ions of Pt (PtCl_6_^2−^). As a result, the L‐ascorbic acid facilitated the reduction of PtCl_6_^2−^ to PtNPs in the presence of absorption on the Au‐ECM and ultimately synthesized the Au@Pt‐ECM. Specifically, the zeta potential measurement verified that the AuNPs at acidic pH conditions had positive surface charges, and UV–vis spectroscopy identified the formation of PtCl_6_^2−^ in the synthesis solution (Figure S11, Supporting Information). After the deposition of PtNPs, as identified in the SEM images (**Figure** **3**A,B), the fibronectin‐templated morphology of the sintered Au‐ECM was maintained, while tiny PtNPs were found on the surface of Au. In the STEM and EDS analyses presented in Figure 3C‒F, a uniformly developed Pt shell was confirmed as encapsulating the interconnected AuNP structures. Furthermore, the diameter of the PtNPs was verified to be ≈3 nm from the HRTEM image collected at the edge of the fibrous structure, as shown in Figure 3G. The measured interplanar spacing value, 0.226 nm, as exhibited in Figure 3G, was assigned to that of the (111) plane for cubic Pt (ICSD No. 76414). In addition, the broadened XRD peaks of Pt validated that the size of PtNPs in the shell of the Au@Pt‐ECM was a few nanometers in scale (Figure S12, Supporting Information).

As the initial proof of concept for the application as an electrocatalyst, the fabricated Au@Pt‐ECM was applied as an electrode to perform the cathodic reaction in electrochemical water splitting, that is, HER. In compliance with previous reports that demonstrated the prominent HER activity of Pt,^[^ ^43^, ^44^, ^45^ ^]^ the PtNPs decorated on the conductive Au‐ECM showed remarkable HER activity in a 0.5 m H_2_SO_4_ electrolyte solution, as illustrated in the polarization curve in Figure 3H, whereas the bare Au‐ECM showed much lower HER activity. In particular, as plotted in the bar graph in Figure 3H, the current density normalized by the electrode area of Au@Pt‐ECM was measured to be 14.4 mA cm^−2^ at an overpotential of 50 mV, which was notably 480 times higher than that of the Au‐ECM (0.03 mA cm^−2^). Au@Pt‐ECM also exhibited a prominent HER activity with a low overpotential of 45 mV at a current density of 10 mA cm^−2^, which is similar to that of the F─SnO_2_@Pt (42 mV),^[^ ^46^ ^]^ Pt_1_/NMHCS (40 mV),^[^ ^47^ ^]^ Pt_1_@Fe─N─C (60 mV),^[^ ^48^ ^]^ and Mo_2_TiC_2_T_x_─Pt_SA_ (30 mV)^[^ ^49^ ^]^ in a 0.5 m H_2_SO_4_ electrolyte solution. The mass activity of HER catalysts based on Pt, which is assessed as the current density normalized by the loaded amount of Pt in the catalysts, is another important parameter for evaluating the electrocatalysts for HER. To measure the mass activity of our biomimetic catalysts, we initially examined the loaded amount of Pt to be 27.9 ± 0.8 µg cm^−2^ (*n* = 3) in Au@Pt‐ECM, which was done by using an inductively coupled plasma mass spectrometer (ICP‐MS). With the measured Pt loading amount, we calculated the mass activity of Au@Pt‐ECM at 50 mV overpotential was 0.517 ± 0.015 A mg^−1^_Pt_ (*n* = 3). In order to compare these results with other research, we summarized the performance values of the other reported catalysts for HER in Table S2 (Supporting Information). For further understanding the HER catalysts, measurements of double‐layer capacitance, from which the electrochemical surface area (ECSA) can be estimated, and the Tafel slope were also carried out for Au@Pt‐ECM (Figure S13, Supporting Information). Particularly, based on the small Tafel slope of Au@Pt‐ECM (34.4 mV dec^−1^), we could estimate its faster HER kinetics than Au‐ECM, which showed a notably large value (490 mV dec^−1^). The Tafel slope of Au@Pt‐ECM also revealed the involvement of the Volmer–Tafel mechanism, as well as the fact that the rate‐determining step is the recombination step.^[^ ^50^ ^]^

In addition, the electrochemical stability of Au@Pt‐ECM was investigated to assess its long‐term operational viability as an electrochemical catalyst. As shown in Figure 3I, cyclic voltammetry measurements were repeated for 500 cycles at a scan rate of 100 mV s^−1^. Even after repeated cycling, we could not observe any noticeable degradation in the catalytic performance of Au@Pt‐ECM from the initial state. The SEM image of Au@Pt‐ECM after 500 cycles of testing confirmed an intact morphology without any PtNP aggregation or detachment (Figure 3J), which corresponds with the electrochemical analysis. Moreover, the chronopotentiometry analysis for Au@Pt‐ECM showed that the overpotential required to reach 10 mA cm^−2^ was shifted by less than 0.1 V over a reaction of 3 h (Figure S14, Supporting Information). These results imply that the dECM‐templated metallic film provided a durable electron‐conducting pathway and anchoring sites for the immobilization of PtNPs during the electrochemical reaction. Overall, the electrocatalyst measurements for our biomimetic materials demonstrated that the high surface area derived from the intrinsic fibronectin architecture, which exhibited a mesh‐like morphology with numerous pores and intertwined fibrillar protein assemblies, was accurately replicated into the Au@Pt‐ECM, even after thermal sintering and additional Pt functionalization, resulting in the prominent catalytic activity and stability of our biomimetic materials in HER.

### 2.3 Synthesis of Pd‐Based Metalized dECMs and Their Utilization as Chemiresistive Hydrogen Gas Sensors

**Figure 4:**
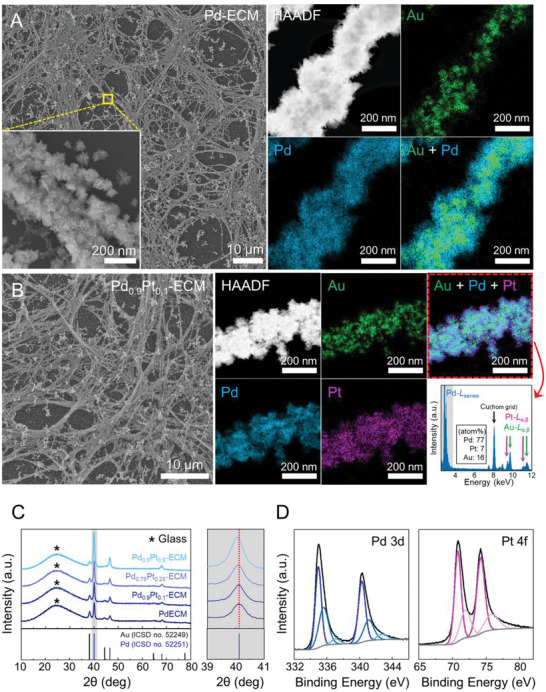
The synthesis and characterization of Pd‐ECM and Pd_x_Pt_1‐x_‐ECM. A) SEM, HAADF, and EDS maps of Pd‐ECM. The inset of the SEM is an enlargement of the yellow box and shows that multipod‐shaped Pd@AuNPs were assembled into a fibrous structure. B) SEM, HAADF, EDS maps, and corresponding EDS spectrum of Pd_0.9_Pt_0.1_‐ECM. C) XRD results of Pd‐ECM and Pd_x_Pt_1‐x_‐ECM (*x* = 0.9, 0.75, and 0.5). As identified in the enlargement of the gray region, a slight diffraction peak shift occurs along the negative 2θ axis at (111) in Pd as the amount of Pt increases. The black star peaks are caused by the glass substrate. D) Electronic state analysis for Pd and Pt composed Pd_0.9_Pt_0.1_‐ECM by XPS.

Our biotemplating method, which utilizes the fibrillar network structure of the dECM as a scaffold, can also be used for fabricating bio‐mimicked architectures by versatile functional metals for application beyond electrochemistry. In this regard, we demonstrated the synthesis of a palladium (Pd)‐based metalized dECM by growing the PdNPs and Pd_x_Pt_1‐x_ alloy NPs, along with the fibronectin assemblies labeled with antibodies. First, AuNPs were grown from secondary antibodies bearing nanogold through just one iteration of the growth step instead of four iterations in the case of Au‐ECM. We then added the negatively charged Pd complex ions together with the L‐ascorbic acid as the reductant. In the same way that the Au@Pt‐ECM was synthesized, this would allow the electrostatic adsorption of the Pd complex ions on the positively charged Au surface and their reduction by L‐ascorbic acid into Pd adjacent to the Au surface. In particular, we used an aqueous 0.5 M NaCl solution as the solvent instead of D.I., which kept the complex ions of Pd derived from Na_2_PdCl_4_·3H_2_O in the form of PdCl_4_^2−^ instead of [PdCl(OH_2_)_3_]^+^ (Figure S15, Supporting Information). As shown in the SEM, STEM, and EDS analyses in **Figure** **4**A, the AuNPs, along with fibronectin, successfully mediated the surface adsorption of the Pd complex ions through electrostatic interaction, resulting in the formation of Au@Pd. It is noted that, when [PdCl(OH_2_)_3_]^+^ was used as the Pd precursor prepared using D.I. as a solvent, we were not able to achieve Au@PdNP configuration because the PdNPs were grown by self‐nucleation (Figure S16, Supporting Information). This result suggests that the electrostatic interaction between AuNPs and Pd precursor is critical for achieving Au@PdNPs. The morphology of the as‐synthesized Au@PdNPs was characterized by a fine multipod surface with an average diameter of 124 ± 30 nm (*n* = 50). Although a few PdNPs showed non‐specific growth via self‐nucleation, as shown in the SEM images, most of the PdNPs were assembled conformally on the fibrous scaffold, accurately following the fibronectin structures.

By incorporating H_2_PtCl_6_ into the growth solution together with the Pd precursor, we could obtain Au@Pd_x_Pt_1‐x_ alloy NP assemblies resembling the fibronectin structures, with controllable Pd‐Pt compositions according to the ratios of each precursor. For example, the metalized dECM displayed in Figure 4B is representative of the case where, compared with the standard protocol for Au@PdNP formation, 10% of the total amount (molar basis) of the Pd precursor used was substituted with Pt precursor. We could achieve a Pd‐Pt alloy system with the addition of only the Pt precursor because H_2_PtCl_6_·6H_2_O would be developed into PtCl_6_^2−^, which also has a negative charge as PdCl_4_^2−^ when dissolved in a 0.5 m NaCl solution (Figure S15, Supporting Information). Therefore, the co‐adsorption of Pt and Pd complex ions on the Au surface was possible, followed by co‐reduction and concomitant growth. The homogeneity of the resultant Pd‐Pt alloy NPs was confirmed by an analysis of the EDS spectrum, indicating that the atomic ratio between Pd and Pt mostly coincides with that of the added precursor. Hereafter, the metalized dECMs prepared by Pd and Pd_x_Pt_1‐x_ alloy NPs are referred to as Pd‐ECM and Pd_x_Pt_1‐x_‐ECM, respectively.

The as‐synthesized Pd_x_Pt_1‐x_‐ECM was characterized in detail through XRD and X‐ray photoelectron spectroscopy (XPS) analyses. Figure 4C shows the XRD patterns of the Pd and Pd_x_Pt_1‐x_‐ECM (*x* = 0.9, 0.75, and 0.5), with characteristic peaks of Au and Pd in a similar appearance. However, the peak located at a little above 2*θ* = 40°, which is shaded in gray and enlarged for visibility, slightly moves toward the lower 2*θ* axis as the percentage of Pt increases in the Pd_x_Pt_1‐x_‐ECM. This phenomenon can be explained by Vegard's law because Pt has a slightly larger lattice constant compared with Pd. Overall, these results validated that the Pd‐Pt alloy NPs were crystallographically uniform throughout the Pd_x_Pt_1‐x_‐ECM. In addition, we carried out XPS to study the chemical states of Pd and Pt in the Pd‐Pt alloy NPs. As plotted in Figure 4D, the binding energies of Pd and Pt in the Pd_0.9_Pt_0.1_‐ECM were examined by analyzing the Pd *3d* and Pt *4f* peaks, which were deconvoluted into three and two pairs of doublets, respectively. As for Pd, the dominant peaks were positioned at 334.95 and 340.3 eV assigned to Pd^0^, while the two pairs of weaker peaks originating from the residual precursor or partial oxidation of Pd^0^ during the drying process were also visible.^[^ ^51^ ^]^ As for Pt, the metallic state Pt^0^ was also dominant in the sense that the highest peaks were 70.8 and 74.25 eV, with the weaker peaks appearing at high binding energies due to being the Pt precursor.^[^ ^52^ ^]^ In particular, both sets of metallic Pd and Pt peaks showed slight peak shifts toward lower binding energies compared with the bulk metals (Pd; 3d_5/2_ = 335.20 eV and 3d_3/2_ = 341.10 eV, Pt; 4f_7/2_ = 71.30 eV, and 4f_5/2_ = 74.50 eV) due to the electronic interactions between Pd and Pt.^[^ ^53^, ^54^ ^]^

**Figure 5:**
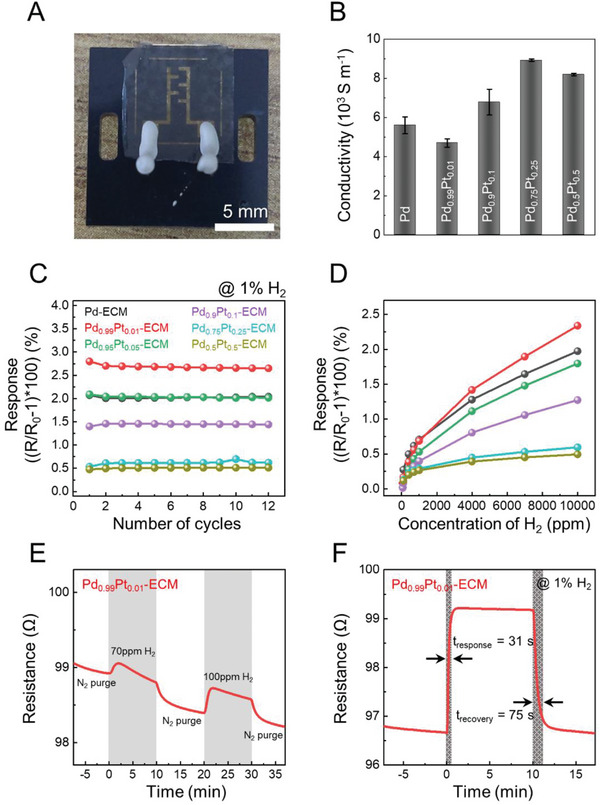
Hydrogen gas‐sensing characteristics of Pd‐ECM and Pd_x_Pt_1‐x_‐ECM. A) Digital image of the manufactured hydrogen sensor. B) The measured electrical conductivity of Pd and Pd_x_Pt_1‐x_‐ECM. Data are presented as the mean ±  SD, *n* = 3. C,D) Results of sensing performance. Cyclic response in 1% hydrogen conditions (C) and response assessment for different concentrations of hydrogen (D) on the fabricated sensors. E) The reliable response in a low hydrogen concentration, 70 ppm, in the case of the Pd_0.99_Pt_0.01_‐ECM. F) Response and recovery times of Pd_0.99_Pt_0.01_‐ECM with ≈1% hydrogen concentration at room temperature.

We then evaluated the hydrogen sensing performance of Pd‐ECM and Pd_x_Pt_1‐x_‐ECM. Metallic Pd and Pd‐based alloys have been widely used as chemiresistors for selective hydrogen sensing applications by virtue of their highly selective reaction with H_2_ to form less conductive PdH_x_, increasing the resistance of the Pd‐based sensing layer that scales with the concentration of H_2_ in a given environment.^[^ ^55^, ^56^ ^]^ Owing to the high surface area and large porosity of the fibronectin architecture originating from the fibrous mesh form, the interfacial surface area between the Pd and H_2_ gas could be maximized for efficient H_2_ sensing. As shown in **Figure** **5**A, we fabricated the chemiresistive sensing device by printing a pair of interdigitated Au electrodes on the metalized dECM using electron beam evaporation. The electrodes were then connected to the data acquisition system using conductive Ag paste. Prior to utilizing the sensing device, we first confirmed that the Pd and Pd_x_Pt_1‐x_‐dECM exhibited conductive properties and demonstrated that electrically conductive pathways were retained in the highly porous dECM‐templated metallic structure for use as a chemiresistor (Figure 5B). In addition, the conductivity of each Pd_x_Pt_1‐x_‐dECM was calculated through the acquired thickness of the films using a surface profile (Figure S17, Supporting Information). The details are summarized in Table S1 (Supporting Information).

In the subsequent gas sensing test, upon exposure to repeated cycles of 1% H_2_ at room temperature, we found that the Pd‐ECM‐based sensors showed stable chemiresistive responses for at least 12 cycles, with an average response of 2.03% and no noticeable degradation in the signals (Figure 5C). As the experimental control, we also prepared an analogous sensing layer by disintegrating the dECM into random pieces of PdNPs via ultrasonication, drop‐casting the resultant PdNP dispersion (Figure S18, Supporting Information) onto the glass substrate, and printing the Au electrode on the surface. This control sample showed abnormal and unstable sensing behavior, likely due to the unwanted junction‐breaking effects between the Pd NPs upon repeated hydrogen‐induced lattice expansion events,^[^ ^57^ ^]^ indicating that the porous dECM architecture played a central role in providing the H_2_‐sensing performance. Moreover, among the samples tested, the Pd_0.99_Pt_0.01_‐ECM‐based sensor exhibited the highest average response of 2.68%, which is higher than that of the Pd‐ECM‐based sensor (2.03%), suggesting that a small amount of Pt could successfully sensitize the H_2_ response of the Pd‐Pt alloy. Interestingly, the involvement of too much Pt (>5 wt.%) degraded the sensor's response, indicating that excess Pt can interfere with the sensing mechanism based on the selective PdH_x_ formation. This observation was confirmed by SEM, STEM, and EDS analyses of the Pd_x_Pt_1‐x_‐ECM with different compositions (Figures S19 and S20, Supporting Information), which showed a decrease in surface roughness and comprehensive coverage of Pt on the outermost layers as more Pt was involved, blocking the Pd from direct exposure to H_2_.^[^ ^58^, ^59^ ^]^

Figure 5D shows that the chemiresistive responses were mostly linear in the range of 10–10 000 ppm H_2_. Notably, the Pd_0.99_Pt_0.01_‐ECM‐based sensor can reliably detect as low as 70 ppm H_2_ (Figure 5E) due to the high porosity of the dECM. Moreover, room‐temperature sensing can be achieved within minutes (31 s to respond and 75 s to recover), supporting the practical viability of the ECM‐based chemiresistive sensor (Figure 5F). Overall, the large surface area architecture of the dECM with high porosity and the Pt sensitization effect of the Pd_x_Pt_1‐x_‐ECM provided the dECM‐based sensors with stable H_2_‐selective sensing properties at room temperature, with a low limit of detection, reasonable sensitivity, and fast response/recovery times. These results demonstrate that the specific protein structure was effectively utilized in the application, leading to remarkable biomimetic hydrogen sensors. In order to compare the hydrogen sensing performances in this study with other research, we summarized the performance values of the existing Pd‐based hydrogen sensors in Table S3 (Supporting Information).

## 3 Conclusion

We have demonstrated the synthesis of metalized thin films with a fibrous architecture by templating the specific protein assembly within the cell‐derived ECM (i.e., fibronectin) using the antibody‐guided biotemplating method. Based on the advantages of antibody‐guided biotemplating, such as specific protein metallization and in situ metal growth from the labeled nanogold, we fabricated a well‐defined fibrous metallic mesh where the densely packed AuNP‐arrays precisely imitated the fibronectin scaffold (Au‐ECM). The Au‐ECM was then thermally sintered to impart electrical conductivity and functionalized with Pt to produce Au@Pt‐ECM, which could be utilized as an electrochemical catalyst for HER. We demonstrated that Au@Pt‐ECM showed prominent HER catalytic activity and stability, verifying that the biotemplated metallic structure properly provided electron‐conducting pathways and anchoring sites for stabilizing PtNPs during the electrochemical reaction. We also confirmed that our biotemplating strategy could be extended to the growth of other types of metals and alloys with compositional control, such as Pd and Pt, on fibronectin protein assembly. The resultant Pd‐ECM and Pd_x_Pt_1‐x_‐ECM can be utilized as a chemiresistor for hydrogen gas sensing due to the high surface area, open porosity, and selective reaction between Pd and H_2_ gas. Compared with our previous work,^[^ ^33^ ^]^ the present study applied a specific protein architecture with a high surface area to relevant applications by considering the structure‐function correlations of the used protein. As a result, we have demonstrated ECM as a universal biotemplate enabling the tailored synthesis of porous metallic nanostructures imitated by specific protein structures for various applications of interest.

There are diverse types of proteins in cell‐derived ECM, whereby their expression quantities and assembled shapes vary along the cells belonging to the muscular, endothelial, or neural systems; these proteins can be targeted with commercially available antibodies.^[^ ^60^, ^61^ ^]^ Therefore, multiple antibodies can be introduced to our approach, followed by metalizing the ECM with different metals grown on each protein type. Furthermore, considering the emergence of techniques for the customizable synthesis of exotic ECM morphologies (e.g., arranged‐ or multiple stacked‐layer ECM^[^ ^27^, ^28^, ^29^, ^62^ ^]^), as well as the potential for the 3D bioprinting of ECM‐protein containing bio‐inks,^[^ ^63^ ^]^ we can anticipate the development of a wider variety of ECM protein structures for use as templates. Metalized ECMs with rationally designed structures were able to broaden their viabilities toward highly delicate applications such as patterned electronic circuits and tissue engineering. In addition to the cell‐derived ECM, the 3D‐specific protein structures from tissue‐ and organ‐derived ECM^[^ ^64^, ^65^ ^]^ can certainly be metalized by antibody‐guided biotemplating as developing the bioengineering techniques related to ultra‐fast and deep immunolabeling.^[^ ^66^, ^67^ ^]^ The combination of our method with such techniques fosters the fabrication of more highly organized and scalable biomimetic structures using tissue‐ and organ‐derived versatile ECMs. As a representative example of the specific protein structures present in organs, we have displayed fluorescence images of several ECM protein structures found in mouse organs, including the liver, heart, and brain (Figure S21, Supporting Information). Furthermore, we achieved super‐resolution volumetric imaging of a mouse brain slice using expansion microscopy (ExM),^[^ ^68^, ^69^ ^]^ as shown in Figure S22 (Supporting Information), where the densely packed fibrous neurites protein structures were clearly distinguished via a multicolor image (Movies S1 and S2, Supporting Information). The fabrication of such enormous and sophisticated structures was challenging using conventional synthetic methods. Therefore, by extending our method to tissue and organ scales, it will become more distinctive and innovative from existing biotemplating techniques.

In applications such as electrocatalysts and chemiresistive sensors, the incorporation of single‐atom or high‐entropy metal nanoparticle fabrication methods, reduction of the interfacial resistance between the electrode and metalized dECM, and modulation of an electronic metal–support interaction into our biotemplating approach are expected to improve its performance.^[^ ^47^, ^48^, ^49^, ^54^, ^70^, ^71^ ^]^ Moreover, antibody‐guided biotemplating can be utilized in numerous applications, especially in biological fields. Although the current study focused on creating electrically conductive biomimetic structures, it is also feasible to introduce inorganic materials showing bionic functionalities, such as antibacterial activities and biomolecule detection.^[^ ^72^, ^73^ ^]^ We are convinced that the high surface area property of dECM can be effectively used in the aforementioned applications.

## 4 Experimental Section

### Materials

L‐ascorbic acid, glycine, chloroplatinic acid hexahydrate (H_2_PtCl_6_·6H_2_O), triton X–100, sodium dodecyl sulfate (SDS), Poly‐L‐lysine, sodium chloride (NaCl), sodium azide, sodium acrylate, acrylamide, *N*,*N*′‐ methylenebis(acrylamide), ammonium persulfate, *N*,*N*,*N*′,*N*′,‐tetramethylethylenediamine (TEMED), 4‐hydroxy‐2,2,6,6‐tetramethylpiperidine‐1‐oxyl (H‐TEMPO), trizma hydrochloride solution, and ethylenediaminetetraacetic acid (EDTA) were purchased from Sigma–Aldrich. Sodium tetrachloropalladate hydrate (Na_2_PdCl_4_·3H_2_O) was purchased from Alfa Aesar. Normal goat serum and normal rabbit serum were purchased from Jackson Immunoresearch, and paraformaldehyde (PFA) and glutaraldehyde (GA) were purchased from Electron Microscopy Sciences. 10× PBS was purchased from Invitrogen. Dulbecco's modified Eagle's medium (DMEM), Dulbecco's phosphate‐buffered saline (DPBS), penicillin‐streptomycin, and fetal bovine serum were purchased from Welgene Inc. Acryloyl‐X, SE (AcX) was purchased from Thermo Fisher and proteinase K was obtained from New England Biolabs. Minimum Essential Medium (MEM) was purchased from Gibco. Deionized water (D.I., 18.2 mω cm) was obtained from a Milli‐Q system. All information about the used antibodies was summarized in Table S4 (Supporting information).

### Cell Culture and Decellularization

Mouse embryonic fibroblast cells (NIH/3T3) from Korean Cell Line Bank were cultured in DMEM supplemented with 10% fetal bovine serum (FBS) and 1% penicillin‐streptomycin at 37°C in humidified 5% CO_2_. A total of 8 × 10^4^ NIH/3T3 cells were seeded on the cover glass, and decellularization proceeded when they reached full confluence. Briefly, the cell sheet was washed with DPBS and the prepared surfactant mixture which was composed of 0.1% SDS and 0.1% triton X‐100 in D.I., was added and kept for a few minutes. Before handling, the surfactant mixture was warmed in a water bath at 37 °C. Followed by decellularization, the resultant dECM was washed with 0.001% Poly‐L‐Lysine in DPBS and fixed using 4% PFA in 1× PBS for 10 min. BS‐C‐1 cells were cultured on Confocal Dish (SPL Life Sciences) in a MEM supplemented with 10% FBS and 1% penicillin‐streptomycin at 37 °C in humidified 5% CO_2_. HEK‐293 cells were cultured on Confocal Dish (SPL) in a DMEM with GlutaMAX supplemented with 10% FBS and 1% penicillin‐streptomycin at 37°C in humidified 5% CO_2_.

### Antibody Staining

The chemically fixed dECM was treated in a quenching solution (0.1 m Glycine in 1× PBS) for 5 min two times and then incubated in a blocking and permeabilization buffer (5% Normal Goat Serum and 0.2% Triton X‐100 in 1× PBS) for 1 h. Thereafter, for labeling the targeted protein in dECM, the dECM was stained with a primary antibody diluted with blocking and permeabilization buffer for 40 min and washed three times using blocking and permeabilization buffer for each 5 min. Then, a secondary antibody diluted with blocking and permeabilization buffer was applied to stained dECM for 40 min, following the two times washing by PBST and one time with 1× PBS for each 5 min. All the experiments proceeded at room temperature and the dilution ratio of each antibody is summarized in Table S4 (Supporting information).

### Preparation of Au‐ECM and Sintering

The dECM that completed the antibody staining was fixed again with the solution (1% GA in 1× PBS) for 10 min, immediately followed by two consecutive washing with PBST and D.I. for each 5 min. The electroless deposition of Au on nanogold conjugated with the secondary antibody by which the targeted protein was labeled was implemented with a solution kit, i.e., GoldEnhance EM kit (Nanoprobes). According to the manufacturer's receipt, the same quantity of four‐component solutions (A: Enhancer, B: Activator, C: Initiator, D: Buffer) were mixed and then applied to stained dECM for 20 min. After that, as‐synthesized Au grown‐dECM was washed three times with D.I. for each 5 min. 300 µL of each component solution was used per a dECM laid on the 1‐inch round cover glass to ensure the experiment's consistency. The additional regrowth step via applying a freshly mixed solution kit was used to obtain further intensified AuNPs over a maximum of four iterations. The synthesized Au grown‐dECM was dried and sintered at 250, 350, or 450 °C in the air for 30 min using an electric furnace (HAN TECH).

### Gold Electrode Deposition and Electrical Conductivity Measurement

To investigate the conductivity of Au‐ECM after the thermal sintering, gold electrodes, which are separated into 200 µm each other, were deposited on a sintered Au‐ECM with a thickness of 150 nm by E‐beam evaporator (KVET‐C500200). Optical microscopy confirmed that the gap between the gold electrodes was uniformly formed without any connections. The current‐voltage (*I‒V*) curves between the gold electrodes deposited on Au‐ECM were recorded using a Keithley 4200A‐SCS with a two‐probe mode. Based on the plotted *I‒V* curve, conductivity (σ) was calculated as below equation.

(1) σ=L·I/T·W·V

In this equation, *L* is the length of the gap between the gold electrodes: 200 µm; *I* is the measured current; *T* is the height of metalized dECM estimated by a surface profiler (Alpha‐Step IQ, KLA‐Tencor); and *W* is the width of the gold electrodes adjacent to the gap: 4 mm; *V* is applied voltage.

### Platinum Growth on Sintered Au‐ECM and Synthesis of Pd‐Based Metalized dECM

To grow PtNPs on sintered Au‐ECM at 350 °C, 100 µL of H_2_PtCl_6_·6H_2_O solution (0.01 m) and 200 µL of L‐ascorbic acid solution (0.1 m) were sequentially added to the sintered Au‐ECM in 2 mL of D.I. The chamber was then placed in an oven at 50 °C for 3 h, and the synthesized Au@Pt‐ECM was washed three times with D.I. For the synthesis of Pd‐ECM or Pd_x_Pt_1‐x_‐ECM (where *x* = 0.99, 0.9, 0.75, and 0.5), 500 µL of Na_2_PdCl_4_·3H_2_O solution (0.01 m, in 0.5 m NaCl) or 500 µL of a mixture of Na_2_PdCl_4_·3H_2_O (0.01 m, in 0.5 m NaCl) and H_2_PtCl_6_·6H_2_O (0.01 m, in 0.5 m NaCl) at a controlled ratio was added to metalized dECM through one iterative Au‐growth in 2 mL NaCl (0.5 m). Next, 1000 µL of L‐ascorbic acid solution (0.1 m, in 0.5 m NaCl) was applied, and the chamber was placed in an oven at 50 °C for 3 h. Finally, the synthesized Pd‐ECM or Pd_x_Pt_1‐x_‐ECM was washed three times with D.I.

### Perfusion, Slicing, Staining, and ExM Process of Mouse Tissues

All procedures involving animals were conducted following the guidelines and approval of the Korea Advanced Institute of Science and Technology Institutional Animal Care and Use Committee (KAIST‐IACUC) under the approval number KA2020–48. Mice (C57BL/6J, ages 8–14 weeks) were housed in a specific pathogen‐free facility at the KAIST Laboratory Animal Resource Center. After anesthetization with isoflurane, mice were transcardially perfused with ice‐cold 1× PBS, followed by ice‐cold 4% PFA in 1× PBS. Whole organs (brain, heart, and liver) were extracted and then kept in the fixation solution (4% PFA in 1× PBS) at 4 °C for 2 h. Fixed organs were sectioned into 100 µm‐thick slices by a vibratome (Lecia VT100S). For the heart, it was embedded in 4% low‐gelling temperature agarose for the sectioning. The sliced organs were incubated with a solution containing 0.1 m glycine and 0.01% sodium azide in 1× PBS to quench autofluorescence. Afterward, all processes associated with antibody staining were performed equally to that implemented on the dECM, except for the incubation time and the used regent volume, which was adjusted as follows: a) blocking and permeabilization: 2 h; b) staining of primary and secondary antibody: 12 h each; and c) washing time: 30 min. When using the rabbit anti‐goat secondary antibody, Normal Rabbit Serum (NRS) was employed instead of NGS. If necessary, 4′,6‐Diamidino‐2‐Phenylindole (DAPI) diluted in 1× PBS was added to the staining solution. The dilution ratio of each antibody is listed in Table S4 (Supporting information). For ExM processing of brain slices, immunostained brain slices were incubated with AcX in 1× PBS at a concentration of 0.1 mg mL^−1^ for 12 h at 4 °C and then washed three times with the 1× PBS. Followed by a gelation solution (7.5% [w/w] sodium acrylate, 2.5% [w/w] acrylamide, 0.15% [w/w] *N*,*N*´‐ methylenebis(acrylamide), 2 m NaCl, 0.2% [w/w] ammonium persulfate (APS), 0.2% [v/v] tetramethylethylenediamine (TEMED), 0.01% [w/w] 4‐hydroxy‐2,2,6,6‐tetramethylpiperidine‐1‐oxyl (H‐TEMPO), 1× PBS) was added at 4 °C for 30 min two times and the slices were placed between two cover glasses with a gelation solution at 37 °C for 2 h. After gelation was completed, the gel was digested by proteinase K (800 units mL^−1^) diluted with 1 mL of a buffer solution (25 mm ethylenediaminetetraacetic acid (EDTA), 50 mm Tris‐HCl [pH 8.0], 0.5% Triton X‐100, 1 m NaCl) at a 1:100 ratio for 12 h with gentle shaking at 37 °C for 6 h. The digested gels were washed in D.I. multiple times until they were fully expanded.

### Characterization

Fluorescence imaging was performed on a Leica DMi8 microscope equipped with an Andor spinning disk confocal microscope system (Dragonfly, Oxford Instruments) through a 40× 1.15 NA water immersion objective. As switching to a bright‐field mode in fusion software, bright‐field images were acquired with the same objective. The detailed morphology of pristine and metalized dECM was measured by a scanning electron microscope (SU8230, Hitachi) at 10 kV. To carry out EDS mapping, another scanning electron microscope (JSM‐IT800, JEOL) mounted EDS (XFlash^®^ FlatQUAD, Bruker) was used. Before SEM imaging, all of the samples were subjected to drying in a 60 °C oven and platinum sputtering to avoid the charge effect. For observation of TEM and STEM, the metalized dECM was peeled off from the cover glass and laid on a holy carbon film Cu grid in D.I. and then dried at a 60 °C oven. TEM/STEM imaging and EDS analyses were progressed with a transmission electron microscope (Talos F200X, Thermo Fisher Scientific) at 200 kV equipped with four integrated silicon‐drift EDS detectors (ChemiSTEM technology). The component and crystallinity of metallic dECM were confirmed by X‐ray diffractometry (SmartLab, RIGAKU) with Cu‐K_α_ radiation. The absorption spectra of metal ion complexes were measured using a UV–vis spectrophotometer (UV‐1900i, Shimadzu), and the zeta potential of AuNPs according to the pH variation was evaluated using an ELS‐Z2000 (Otsuka). The surface chemical state of metalized dECM was investigated by X‐ray photoelectron spectroscopy (K‐alpha, Thermo Fisher Scientific) with a monochromatic Al‐Kα X‐ray source and flood gun emission of 150 µA. To measure the compositional amount in the metalized dECM, an inductively coupled plasma mass spectrometer (iCAP RQ, Thermo Scientific) was applied after pretreatment of a 200 °C microwave reaction over 30 min in a mixture of 70% HNO_3_ and 30% HCl.

### Electrochemical Measurements

All electrochemical measurements were conducted with a potentiostat (Biologic SP‐300) in a 0.5 m H_2_SO_4_ solution evaluated at ≈0.3 pH. A graphite rod was used as a counter electrode and Ag/AgCl (3 m KCl) reference electrode was used. The measured potential values were calibrated with respect to the reversible hydrogen electrode (RHE) by the following equation at 25 °C,

(2) $$E_{\mathrm{RHE}}=E_{\mathrm{Ag}/\mathrm{AgCl}}+0.059\cdot \mathrm{pH}+E{}_{\mathrm{Ag}/\mathrm{AgCl}}^{\circ }$$

where *E*_RHE_ is the calibrated potential as to RHE, *E*_Ag/AgCl_ is the measured potential, and *E*°_Ag/AgCl_ is the standard potential of Ag/AgCl (3 m KCl) at 25 °C, i.e., 0.21 V. To fabricate the working electrode from metalized dECM, the cover glass layered with metallic dECM was sliced into the similar area (≈0.2 cm^2^) and connected with copper wire using the silver paste, and finally covered with inert epoxy resin (EP‐04), only exposing the metalized dECM. The cyclic potential was applied to the samples at a rate of 10 mV s^−1^ between −0.17 and 0.3 V versus RHE without the uncompensated resistance (*R*) correction. Electrochemical impedance spectroscopy (EIS) was also performed at a frequency range from 10 Hz to 1 MHz with an amplitude of 10 mV to estimate the uncompensated resistance (*R*) and used these values for *iR* correction. The electrochemical double‐layer capacitance of the working electrodes was measured to examine the catalytic active surface area by recording the current values in a range from 0.07 to 0.17 V versus RHE as a function of scan rate.

### Fabrication of the Chemiresistive Hydrogen Sensor and Evaluation of Hydrogen Sensing Properties

The dECM‐based hydrogen sensor was fabricated directly from the Pd and Pd_x_Pt_1‐x_‐ECM films on the glass substrate. To produce the sensor, the metalized dECM film was covered with a negative mask and sputtered with Au using an electron beam evaporator (SNTEK VER‐5004, EQCELL Co., Ltd., Suwon, Korea) to fabricate the 100 nm thick Au interdigitated electrode pattern on top of the metalized dECM film. Each electrode was electrically interfaced by conductive silver paste to a metal pin, which could be plugged into our homemade gas sensor testing equipment for dynamic resistance measurements across the metalized dECM film. Our homemade equipment consisted of a data acquisition system with a 16‐channel multiplexer (34972A and 34902A, Agilent Technologies, Inc.) as well as a gas flow regulation system based on multiple mass flow controllers (VIC‐D210, MKP Co., Ltd., Hwaseong, Korea). The concentration of H_2_ was controlled by modulating the gas flow rates from the 1% H_2_ bombe (N_2_ background) in a specific ratio to that of the pure N_2_ bombe, while maintaining a total overall flow rate of 1000 sccm. Before the experiment, the sensors were first purged with pure N_2_ for at least 1 h for stabilization. In a typical measurement cycle, the sensors were exposed to a flow of H_2_/N_2_ gas at a given concentration for 10 min to allow monitoring of the resistance change, followed by pure N_2_ for another 10 min for recovery. Subsequent cycles would be conducted with concentrations of H_2_ ranging from 0.001% H_2_ to 1% H_2_. The changes in the resistance of the sensors were monitored in a 4‐s interval throughout the experiment. In the end, the resistance data was converted into the response data for each concentration of H_2_ by normalizing the change in the resistance value upon exposure to H_2_/N_2_ to the baseline resistance value (i.e., in a pure N_2_ environment immediately prior to exposure to the target gas). The response and recovery times were reported as the time elapsed to achieve 90% of the resistance change, either increasing or decreasing, respectively.

### Image and Statistical Analysis

Unless otherwise stated, fluorescence images were projected using Image J's maximum intensity projection (Image > Stacks > Z project) to display the overall protein structure within the cell‐ or organ‐derived ECM. For the *z*‐stack images shown in Figure S22 and Video S1 (Supporting Information), all the *z*‐stack images were subjected to intensity normalization, compensating for the decrement of intensity value that generally occurs as the depth of the focal plane increases due to antibody diffusion issues. Specifically, the mean intensity value of the first *z*‐stack image, expected to have the highest intensity, was divided by the mean intensity value of the given *z*‐stack image. Then, the intensity of the corresponding *z*‐stack image was multiplied by the resultant value. Finally, each *z*‐stack image was further normalized by dividing its maximum intensity value. All the intensity normalization processes were performed using MATLAB R2020b software. Quantitative data (e.g., mean ±  SD) was calculated using Microsoft Excel 2019.

## Conflict of Interest

The authors declare no conflict of interest.

## Supporting information

Supporting Information

Supplemental Movie 1

Supplemental Movie 2

## Data Availability

The data that support the findings of this study are available from the corresponding author upon reasonable request.
