# Supplementary material for: Metallization of Targeted Protein Assemblies in Cell‐Derived Extracellular Matrix by Antibody‐Guided Biotemplating
Source: Adv Sci (Weinh). 2023 Oct 18;10(35):2302830. doi: 10.1002/advs.202302830 (PMC10724409; doi:10.1002/advs.202302830)
Supplement: Supplementary file 1 — Supporting Information [file ADVS-10-2302830-s002.pdf]

ADVANCED SCIENCE
Open Access

Supporting Information

for *Adv. Sci.*, DOI 10.1002/advs.202302830

Metallization of Targeted Protein Assemblies in Cell-Derived Extracellular Matrix by Antibody-Guided Biotemplating

Chang Woo Song, Jaewan Ahn, Insung Yong, Nakhyun Kim, Chan E Park, Sein Kim, Sung-Yoon Chung, Pilnam Kim, Il-Doo Kim and Jae-Byum Chang**

Supporting Information

Metallization of Targeted Protein Assemblies in Cell-Derived Extracellular Matrix by Antibody-Guided Biotemplating

Chang Woo Song, Jaewan Ahn, Insung Yong, Nakhyun Kim, Chan E Park, Sein Kim, Sung-Yoon Chung, Pilnam Kim, Il-Doo Kim, and Jae-Byum Chang**

Supplemetary Movies

Movie S1. Confocal microscopy z-stack image after expansion.

Movie S2. 3D visualization of the z-stack image shown in Movie S1.

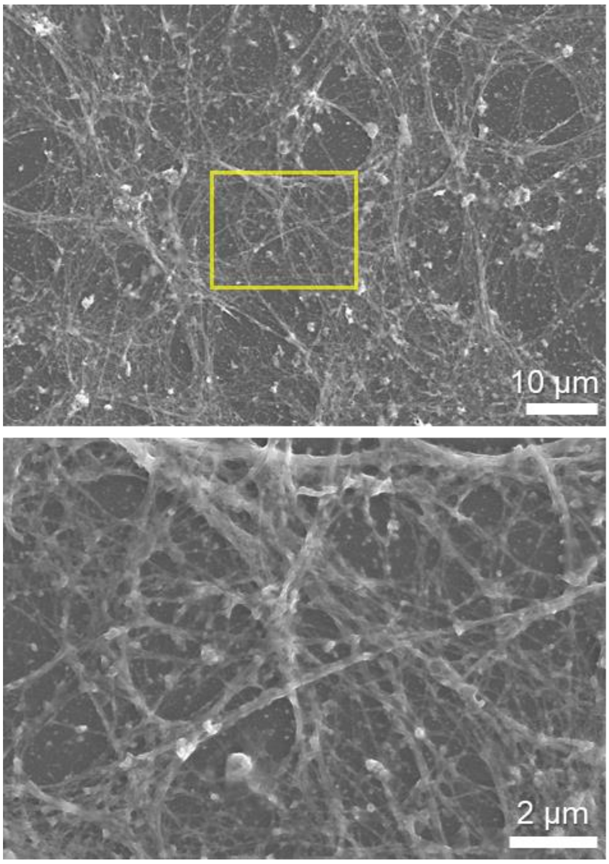

Figure S1. SEM images of ECM after decellularization. Negative staining with 2% uranyl acetate was applied to highlight the dECM in an electron microscope. The bottom is a magnified image of the yellow box.

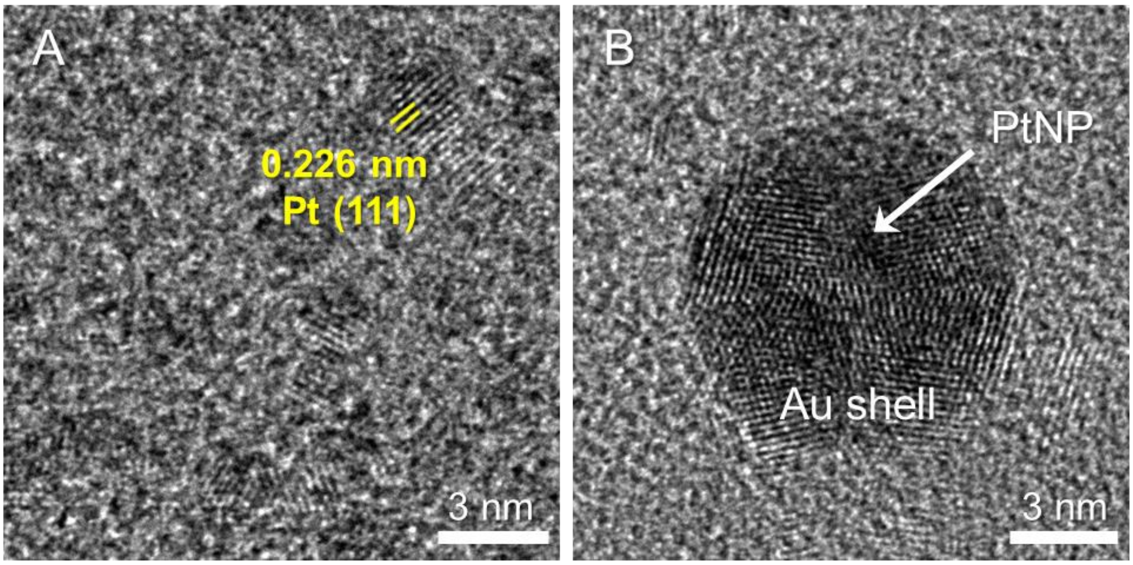

Figure S2. Confirmation of AuNP development on different metal NPs acting as seeds using high-resolution TEM images. (A) PtNPs (Sigma-Aldrich, 773875) with a diameter of about 3 nm. (B) AuNP was grown on PtNP through the catalytic reduction effect of a metal surface. Approximately a 9 nm-diameter Pt@AuNP was formed by a 3-minute gold growth process.

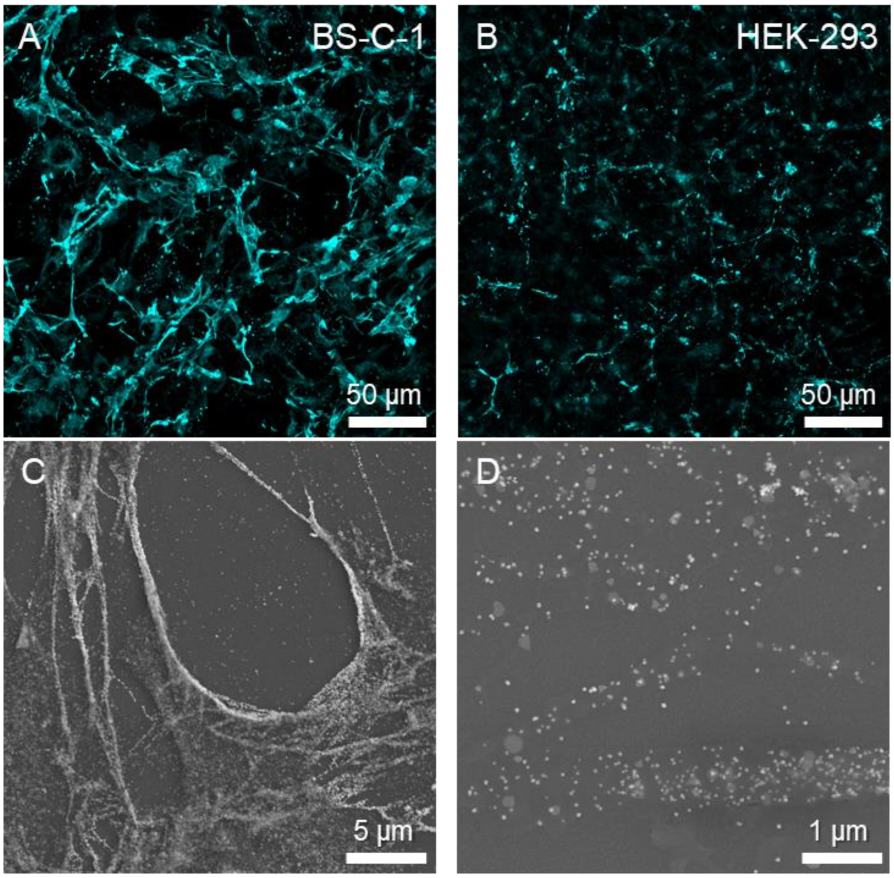

Figure S3. Fluorescence images of fibronectin structures in different cell lines and SEM images of metallic structures mimicking them through antibody-guided biotemplating. (A, C) The observed fibronectin structures were derived from monkey kidney epithelial cells (BS-C-1) and (B, D) human embryonic kidney cells (HEK-293). We performed the experiments without decellularization when each type of cell reached full confluency.

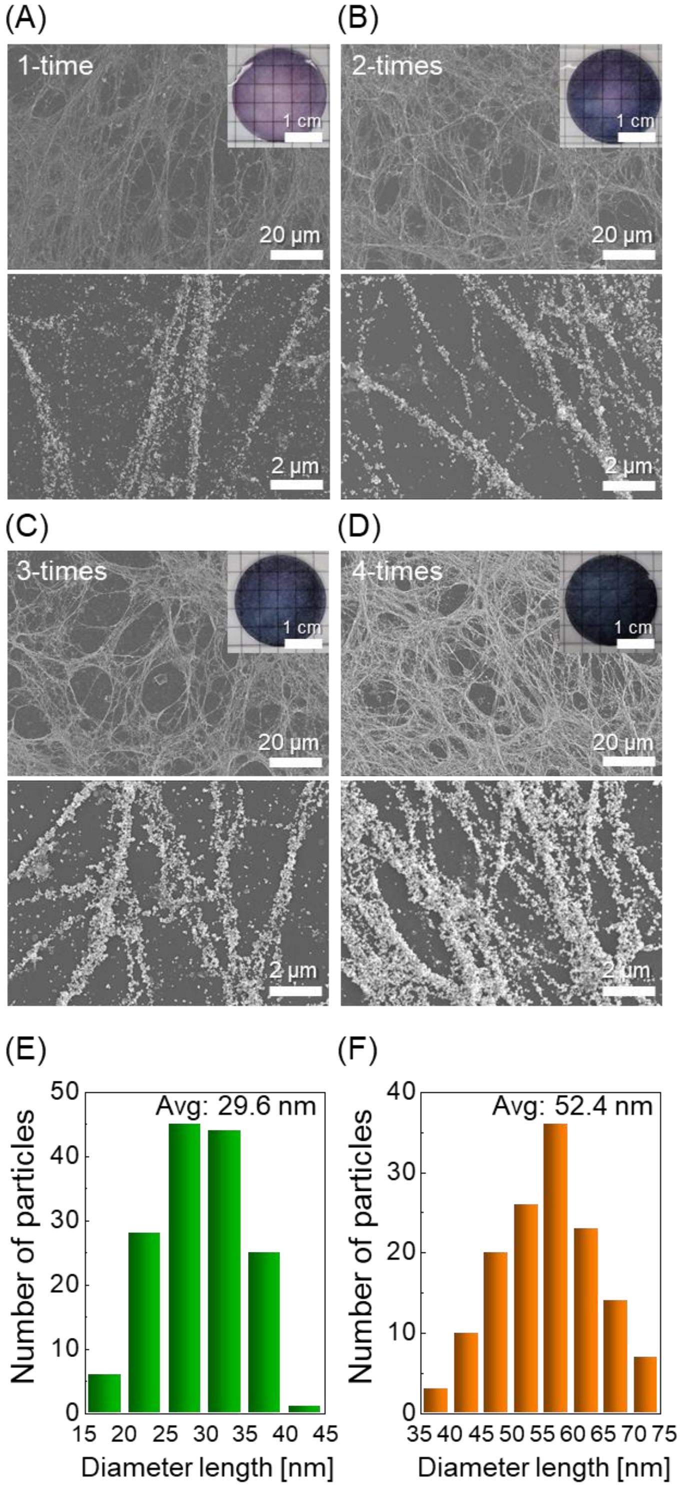

Figure S4. The size variation of grown AuNPs per the number of Au growth. (A–D) Four pairs of SEM images of metalized dECM along with fibronectin from one to four times Au growth. As shown in the inset digital images, the color of the metalized dECM darkened as the number of Au growth increased. (E–F) Diameter distribution of AuNPs after one (E) and four (F) times of Au development.

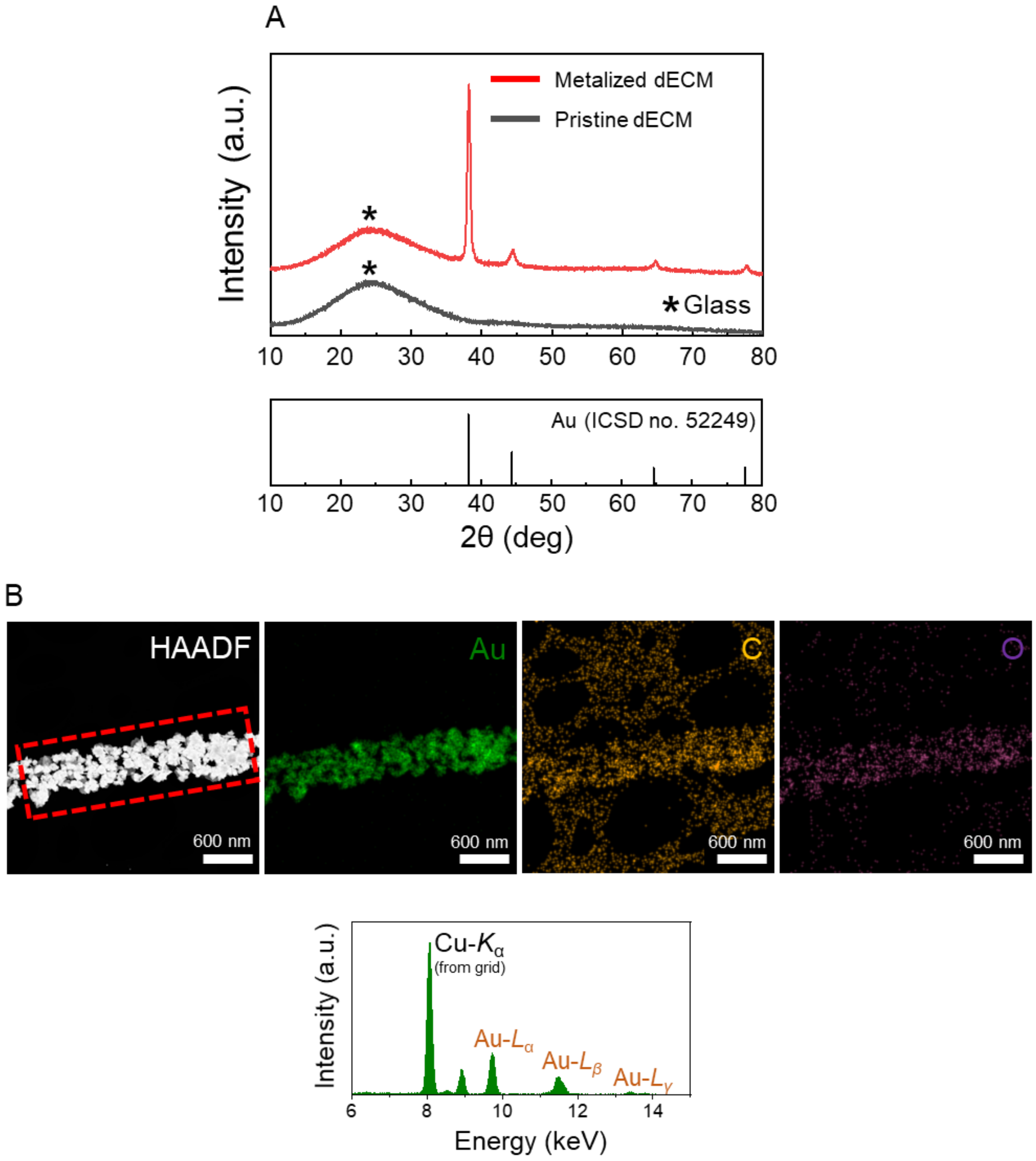

Figure S5. Compositional analysis of synthesized dECM after Au growth. (A) XRD patterns of the metalized dECM and pristine dECM. (B) High-angle annular dark-field (HAADF) image, EDS map, and corresponding spectrum of a single strand AuNPs-array that imitated the fibronectin structure.

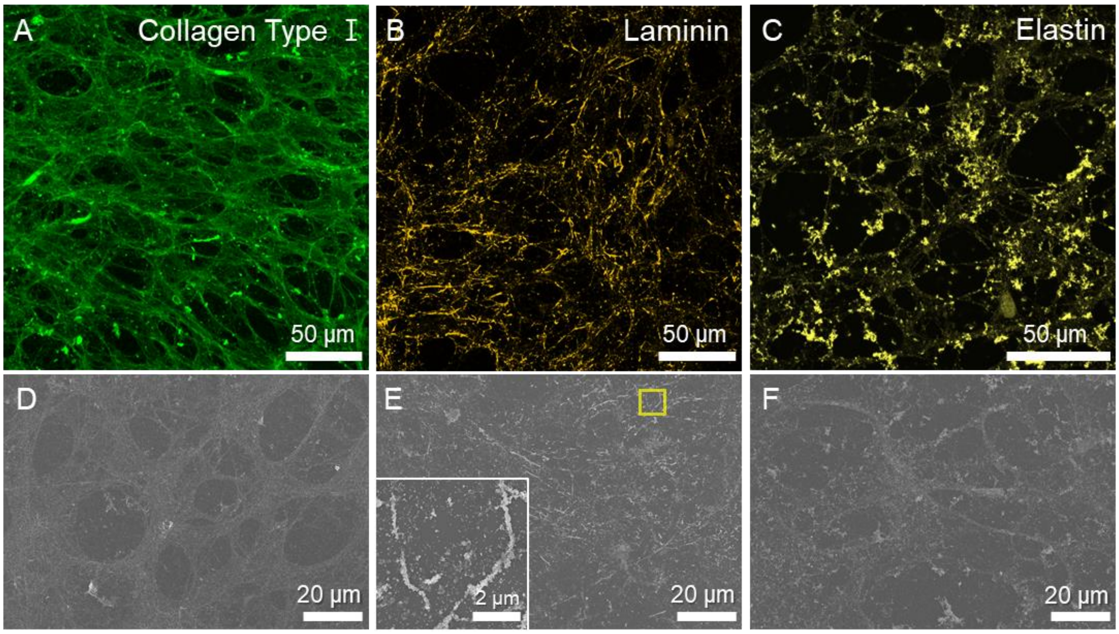

Figure S6. Fluorescence images showing the structures of three different types of proteins made of dECM and SEM images of AuNP-assemblies templated by each protein. (A, D) Collagen type I was found to have an intertwined fibrous mesh structure with micro-sized pores, similar to fibronectin in the fluorescence image, but AuNP-fibrous assemblies were identified with a low labeling density of AuNPs in the SEM image. (B, E) Laminin exhibited disconnected structures in both the fluorescence and SEM images. (C, F) Elastin also showed a fibrous structure in the dECM, but the fluorescence images showed a weak signal, which was corroborated by the low labeling density of AuNPs in the SEM image.

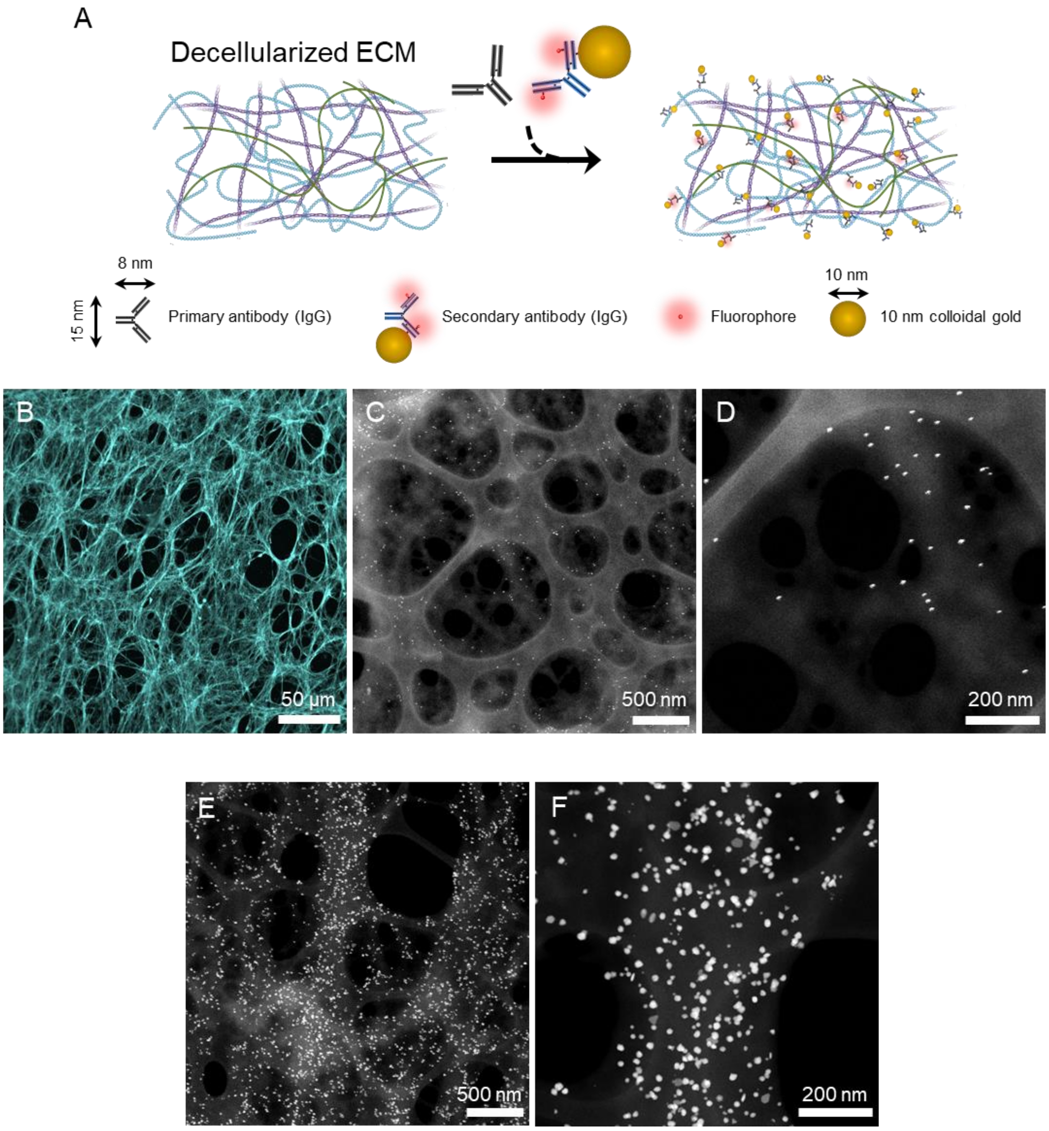

Figure S7. Comparison of the labeling density according to the size of AuNPs conjugated with the secondary antibody. (A) Illustration of an experiment using a secondary antibody-bearing 10 nm colloidal AuNPs. (B) In the fluorescence image, the fibronectin structure was clearly visible in the use of the secondary antibody-bearing 10 nm colloidal AuNPs. (C, D) Nanoscale measurements using STEM verified that the labeling density of the 10 nm colloidal AuNPs was insufficient due to the low staining efficiency of the secondary antibody containing the 10 nm colloidal AuNPs. (E, F) In contrast, a high labeling density of AuNPs was ensured using secondary antibody-bearing nanogold. To accurately compare with (C, D), slight Au growth was applied to the nanogold, growing their size to about 15 nm, and measurements were taken at the same magnification as in (C, D).

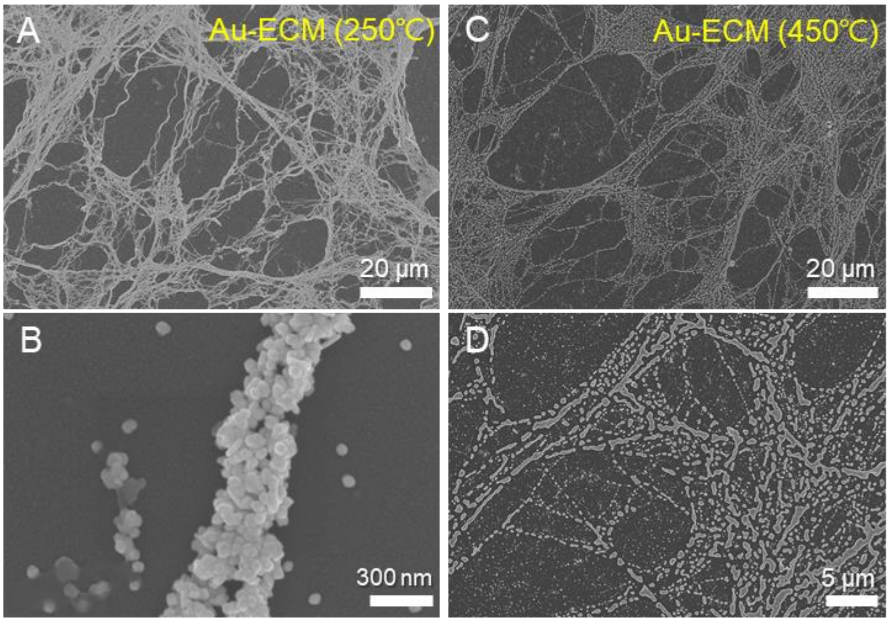

Figure S8. (A, B) Low and high magnification SEM images of Au-ECM sintered at 250 ℃. (C, D) Low and high magnification SEM images of Au-ECM sintered at 450 ℃.

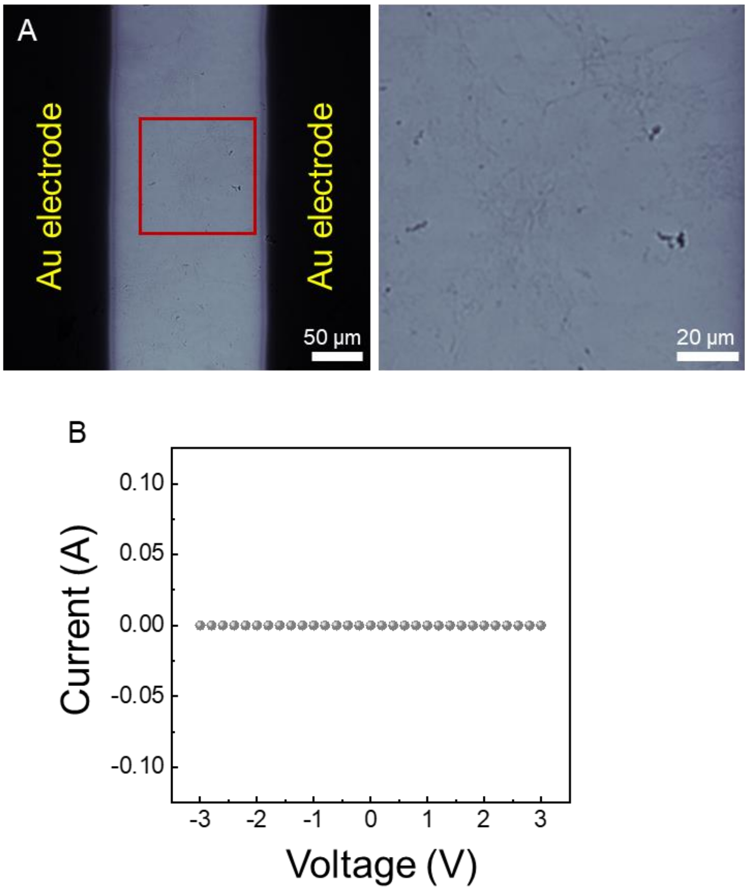

Figure S9. Electrical conductivity of dECM treated with thermal sintering at 350 ℃. (A) Bright-field images of the interface region between the Au electrodes (left panel), and an enlargement of the red box (right panel). (B) The current-voltage curve of thermally sintered dECM.

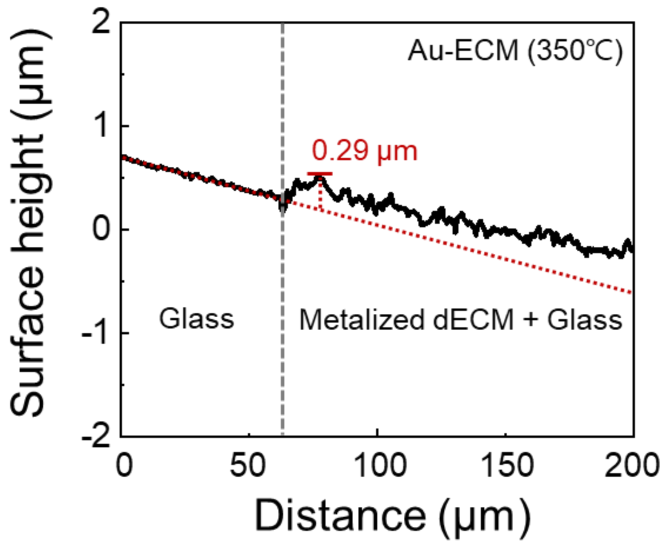

Figure S10. Measurement of the thickness of Au-ECM sintered at 350 ℃ using a surface profiler. The fluctuating graph originated from the fibrous structure of metalized dECM.

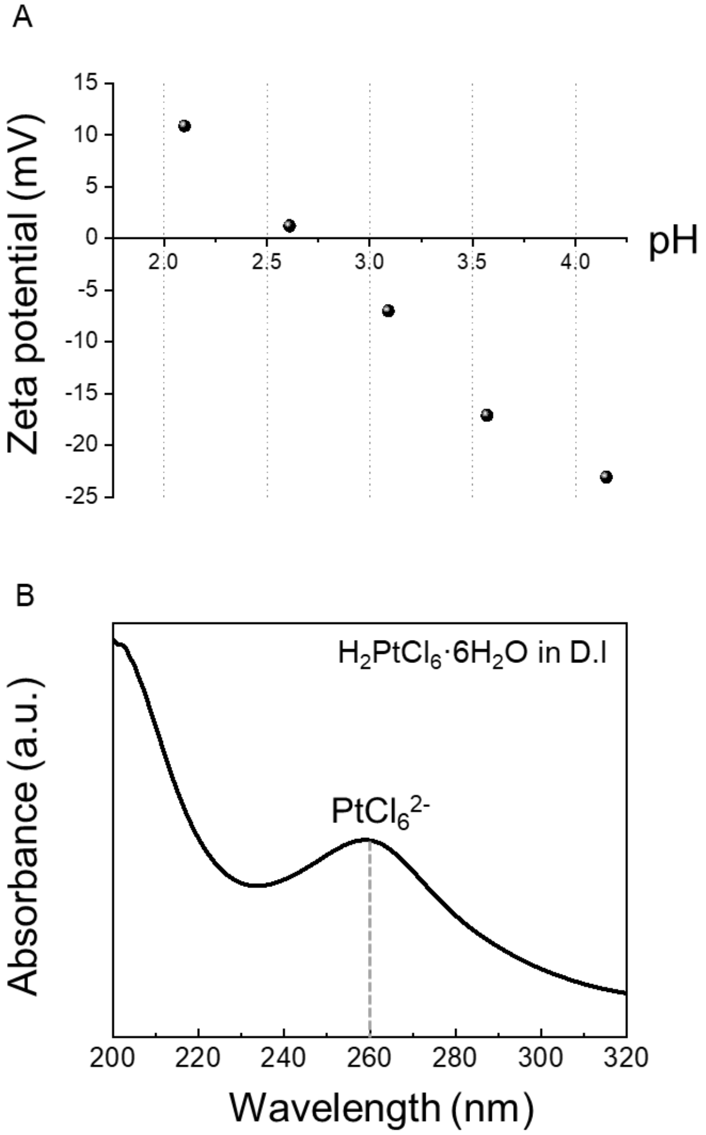

Figure S11. Zeta potential measurement of AuNPs and UV-Vis spectrum of $H_2PtCl_6{\cdot}6H_2O$ in D.I. (A) The zeta potential of AuNPs was measured as a function of solution pH, revealing that the AuNPs had a positive charge in the synthesis condition. The AuNPs used to measure the zeta potential were obtained through electroless plating of Au on a 1.4 nm nanogold not conjugated with antibodies (Nanoprobes, #2025). (B) The generation of $PtCl_6^{2-}$ was verified by the appearance of peaks at wavelength of 200 and 260 nm.[1]

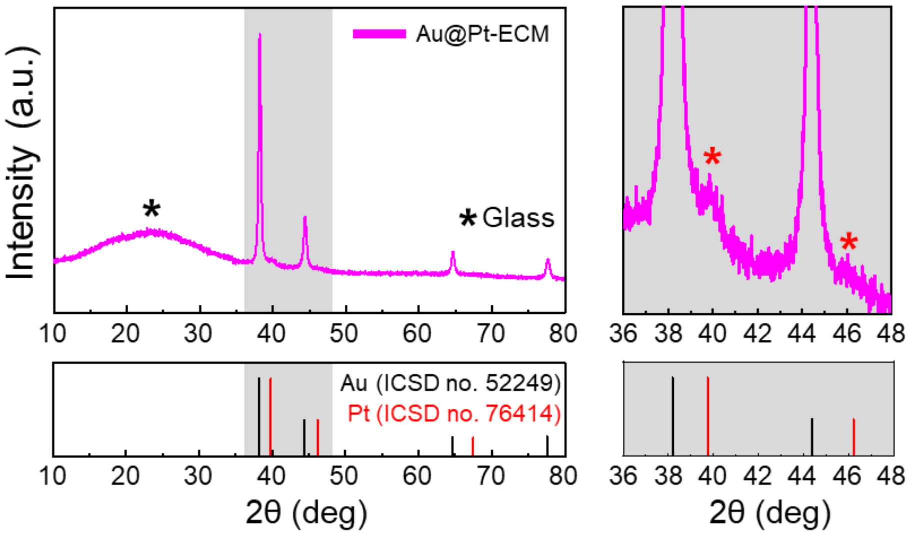

Figure S12. XRD pattern of Au@Pt-ECM. The weak peaks marked by red stars indicated that a Pt shell was formed.

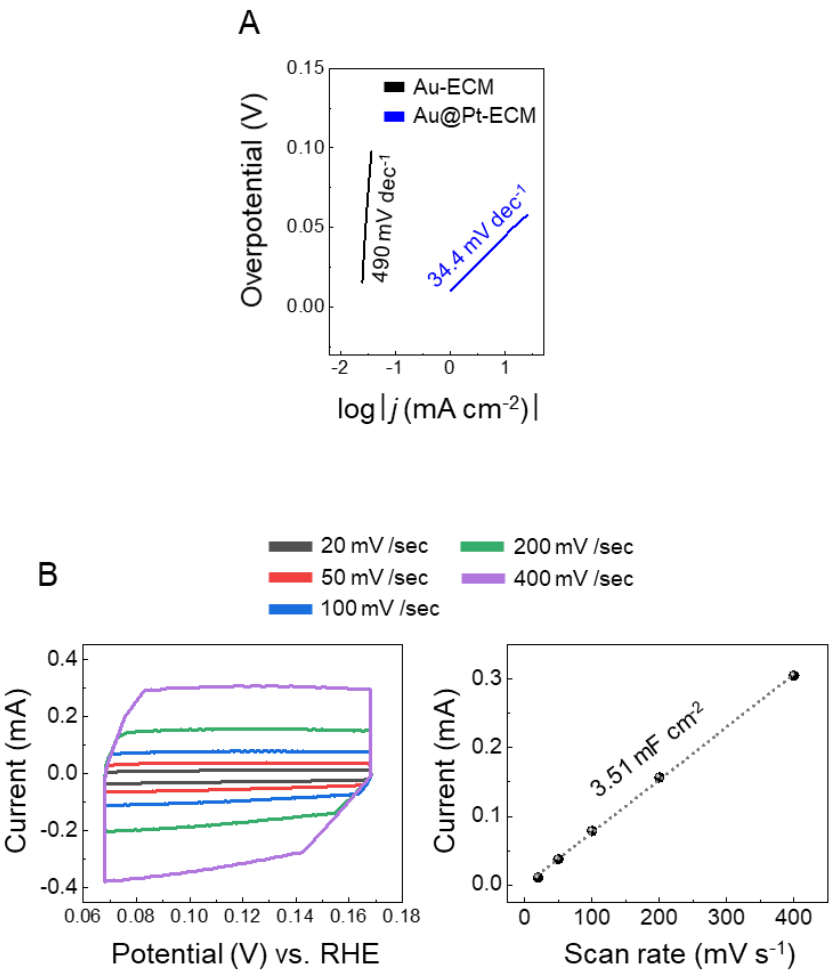

Figure S13. Tafel plot and cyclic voltammograms for double-layer capacitance evaluation. (A) The Au@Pt-ECM exhibited a lower Tafel slope (34.4 mV dec^{-1}) than that of Au-ECM (490 mV dec^{-1}), implying that Au@Pt-ECM has a favorable reaction kinetic for HER than Au-ECM. (B) Cyclic voltammograms were acquired in a non-faradaic region at different scan rates using Au@Pt-ECM (left panel). By plotting the anodic current at 0.12 V (vs. RHE) as a function of scan rate, the double-layer capacitance was calculated (right panel) to be 3.51 mF cm^{-2}.

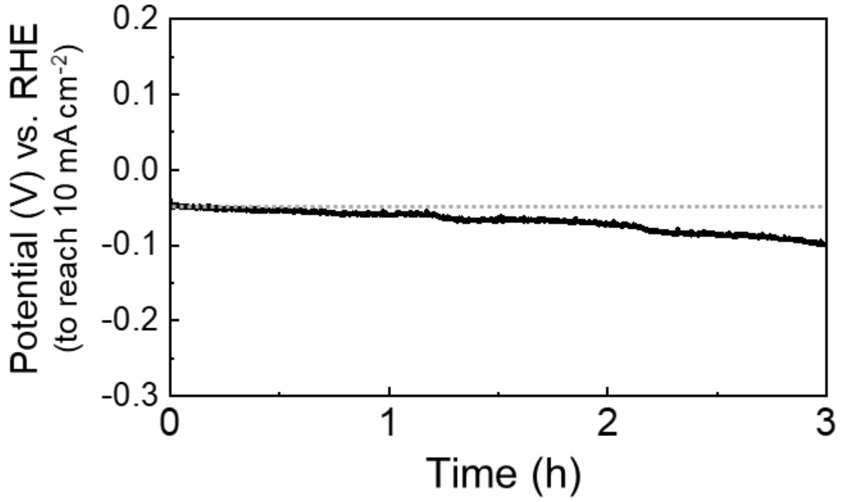

Figure S14. The chronopotentiometry measurement of Au@Pt-ECM to acquire 10 mA cm^{-2} as a function of time.

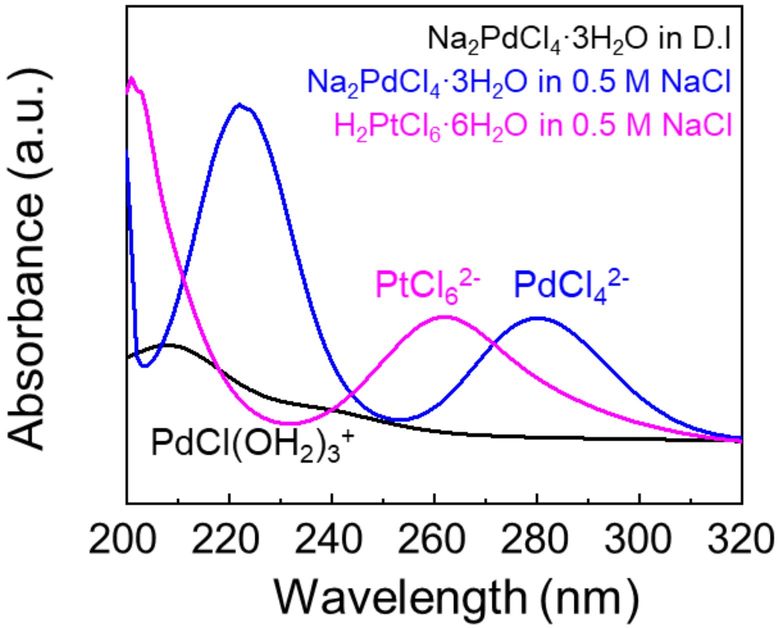

Figure S15. Identifying complex ions by UV-Vis spectroscopy. $[PdCl(OH_2)_3]^+$ has a reference peak at 211 nm, while $[PdCl_4]^{2-}$ has reference peaks at 222 and 280 nm. $[PtCl_6]^{2-}$ exhibits absorption peaks at 200 and 260 nm wavelengths.[1][2]

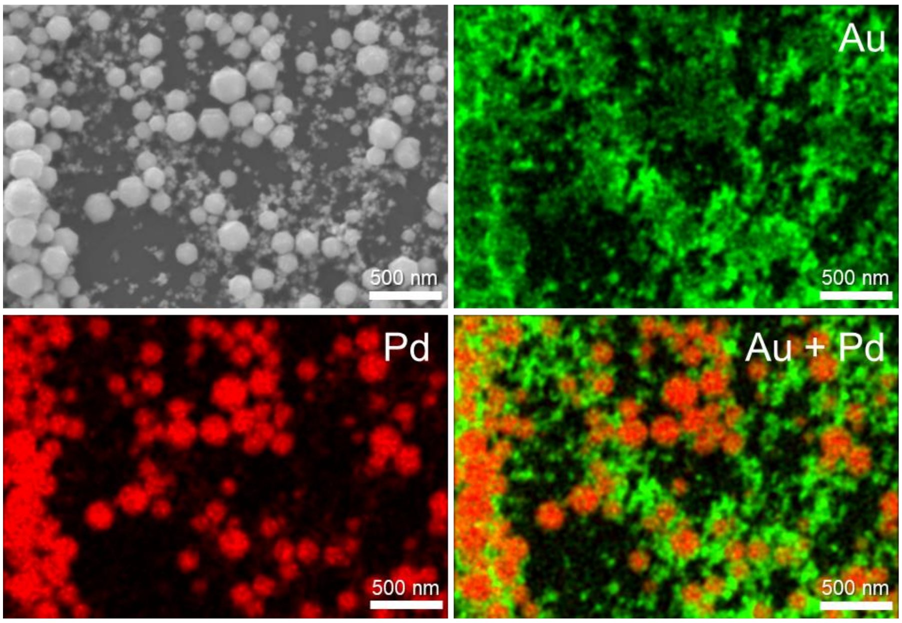

Figure S16. As observed by SEM and EDS maps, the formation of Au@PdNP could not be achieved when $[PdCl(OH_2)_3]^+$ was used as a Pd precursor due to the electrostatic repulsion between the positively charged AuNP surface and positively charged Pd complex ions.

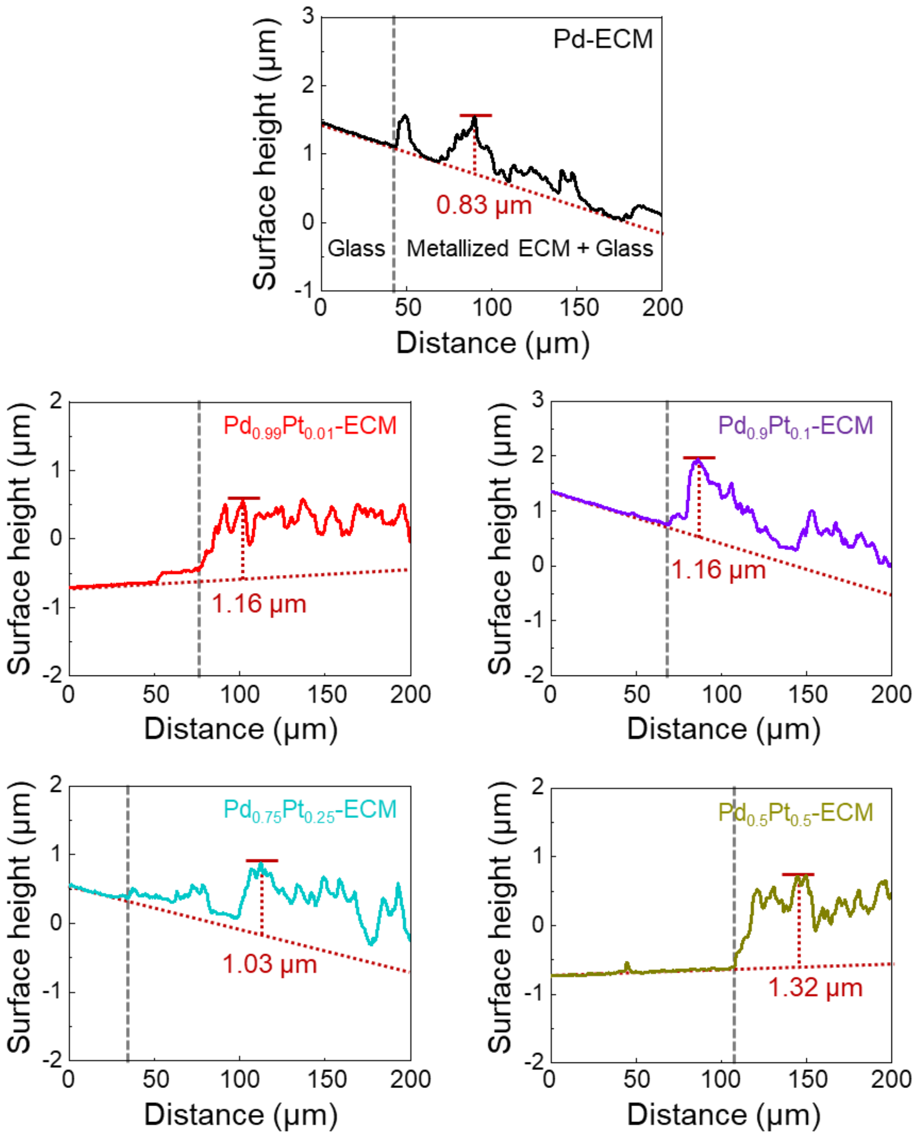

Figure S17. Measurement of the thickness of Pd-ECM and Pd_xPt_{1-x}-ECM using a surface profiler.

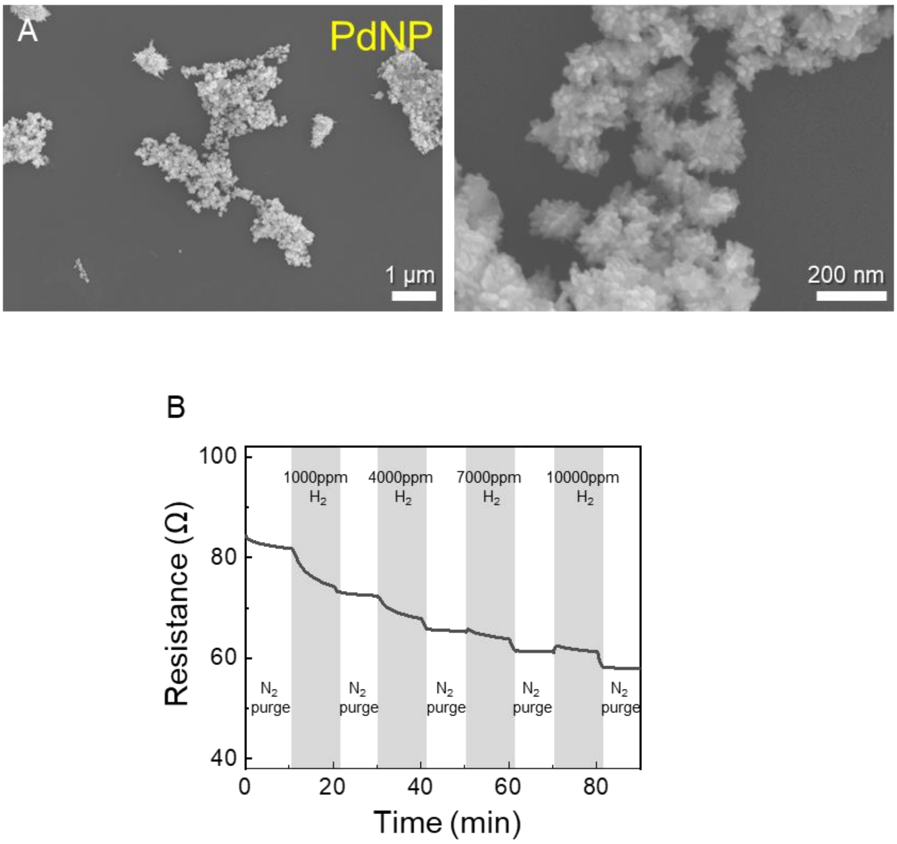

Figure S18. The dissociated PdNPs from Pd-ECM and their hydrogen gas sensing performance. (A) SEM images of the dissociated PdNPs. (B) Response results of the drop-casted PdNPs on sensing substrate toward different concentrations of hydrogen.

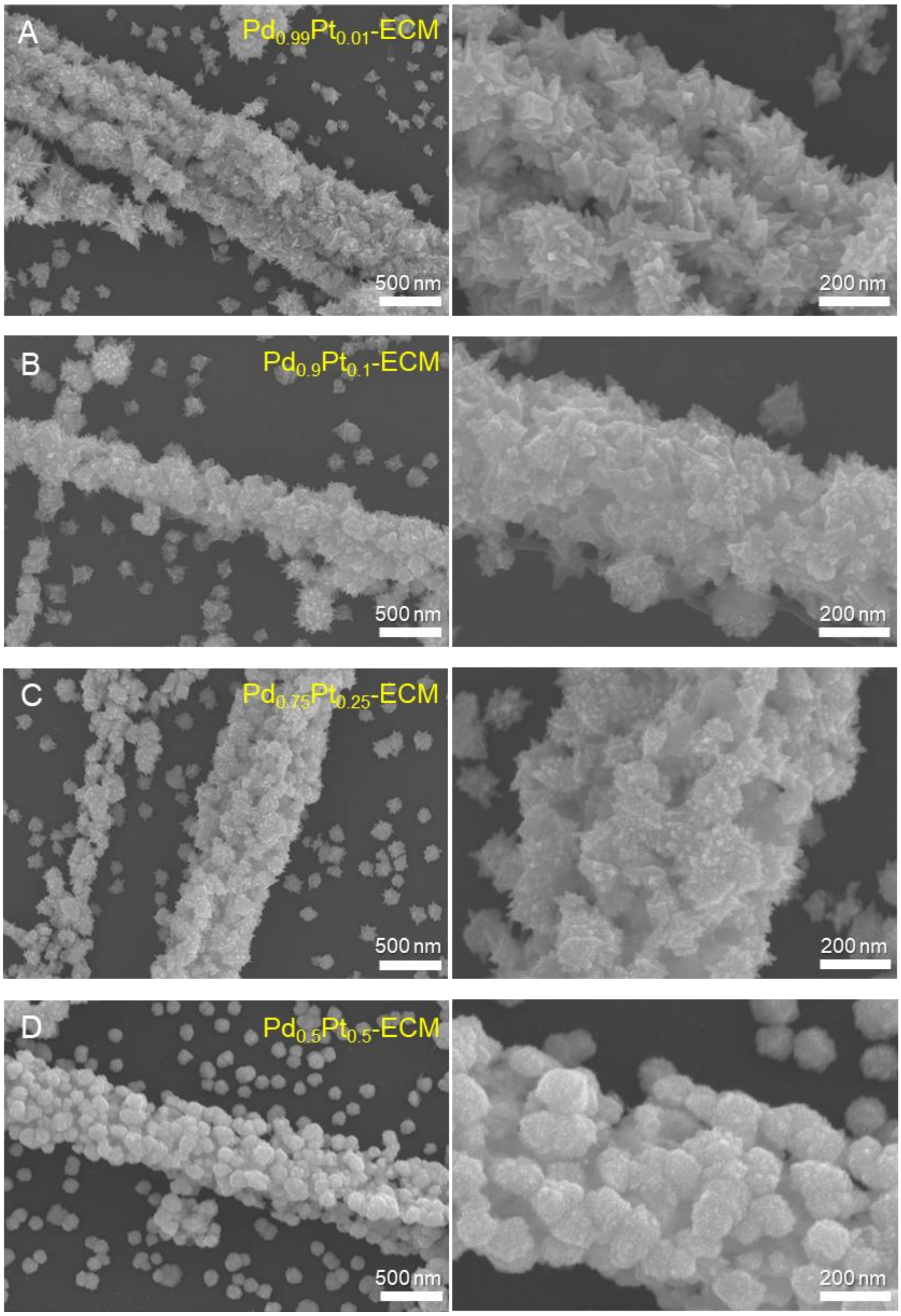

Figure S19. The morphology variation of Pd-Pt alloy NPs as a function of the amount of added Pt. As manifested in SEM images, the surface roughness decreased as the percentage of Pt increased, leading to a reduction of hydrogen sensing performance. (A) $Pd_{0.99}Pt_{0.01}$-ECM, (B) $Pd_{0.9}Pt_{0.1}$-ECM, (C) $Pd_{0.75}Pt_{0.25}$-ECM, and (D) $Pd_{0.5}Pt_{0.5}$-ECM

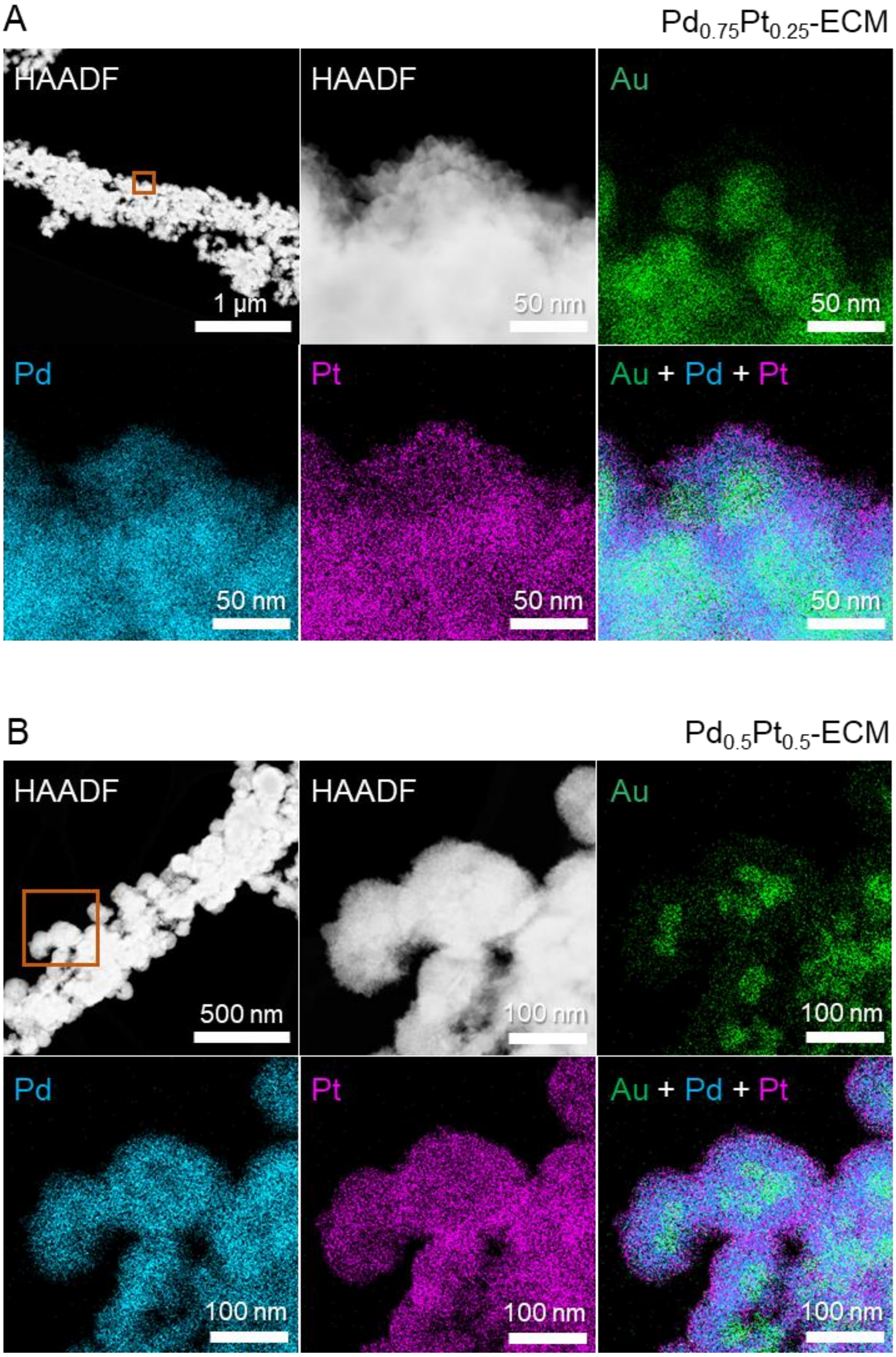

Figure S20. The generation Pt layer on the outermost PdPt alloy NPs as the percentage of Pt increased. (A) HAADF images and EDS composition maps for $Pd_{0.75}Pt_{0.25}$-ECM. (B) HAADF images and EDS composition maps for $Pd_{0.5}Pt_{0.5}$-ECM. The interference with the reaction between H_2 and Pd by the formed Pt layer would decrease the sensing capability. The dark orange areas were observed through EDS analysis.

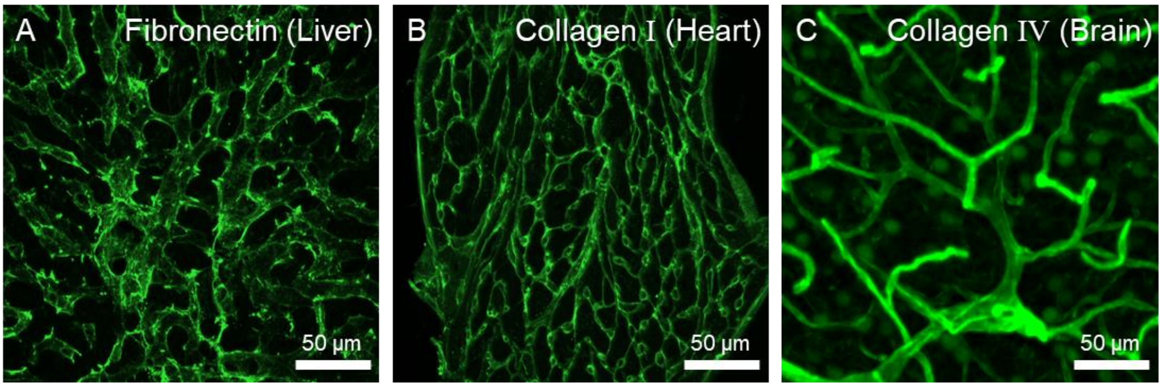

Figure S21. Fluorescence images of several ECM protein assemblies in different mouse organ slices. As illustrated in the fluorescence images, a variety of morphologies, including (A) vascular, (B) porous, and (C) fibrous structures, existed in mouse organ-derived ECM.

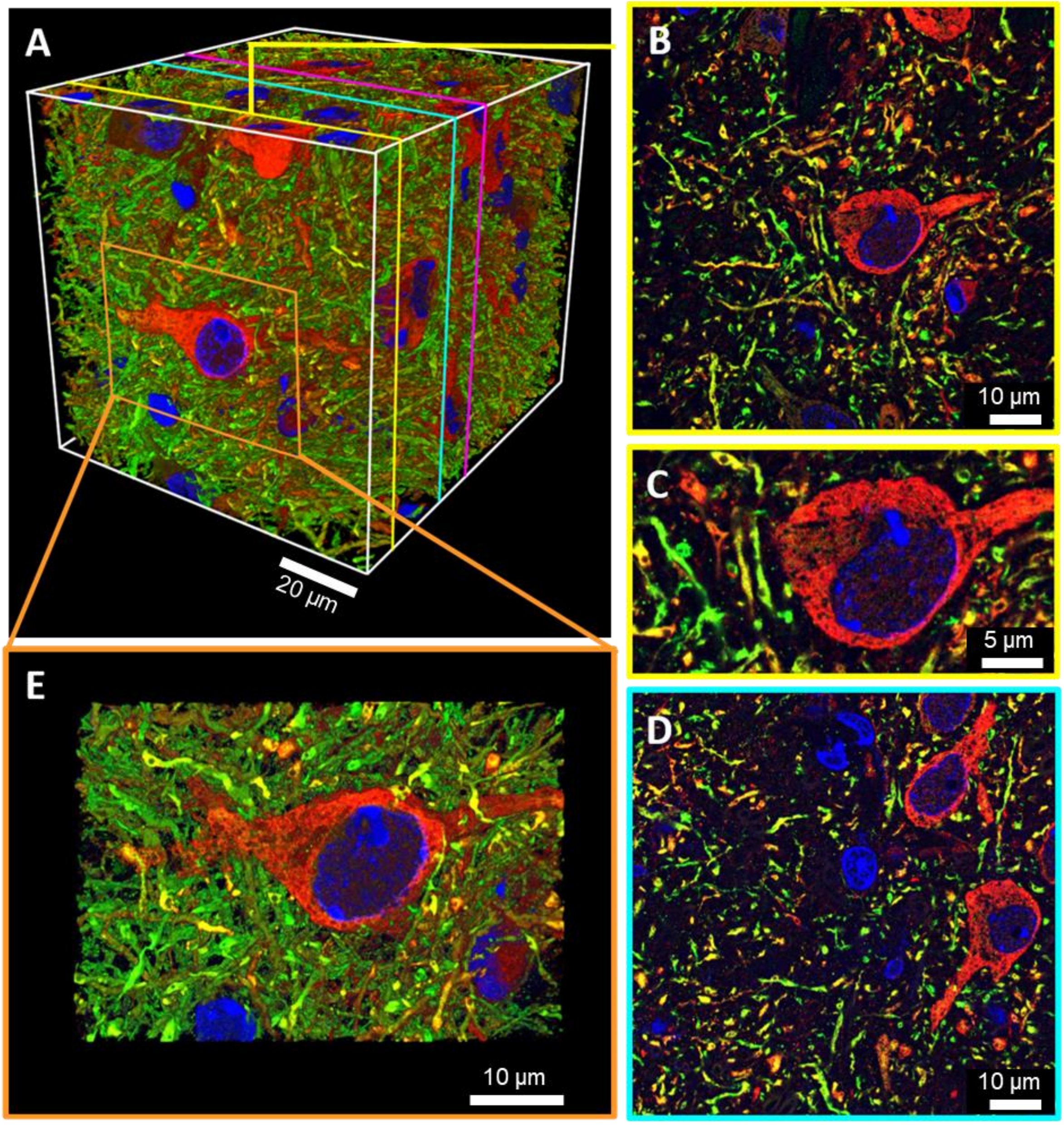

Figure S22. Super-resolution volumetric imaging of mouse brain slices via expansion microscopy. (A) 3D view of an expanded mouse brain slice (blue: DAPI, red: TH, green: MAP2). The size of the image was 82 µm by 82 µm by 80 µm before expansion. (B–D) Single-plane confocal microscopy image of the focal planes of the specimen shown in (A). (E) Magnified view of the 3D visualization in (A). The length scales are presented in pre-expansion dimensions. DAPI: 4′,6-Diamidino-2-Phenylindole; TH: Tyrosine Hydroxylase.

Table S1. Summary of the electrical conductivity of metalized dECM in this study. Data are presented as mean ± SD, $n = 3$ (each independent experiment).

**Sample**	**Au-ECM**	**Au-ECM (after 250 ℃ sintering)**	**Au-ECM (after 350 ℃ sintering)**	**Au-ECM (after 450 ℃ sintering)**
Conductivity ($S\ m^{-1}$)	$3.59 \pm 0.14 \times 10^{-3}$	$2.21 \pm 0.32 \times 10^{2}$	$3.46 \pm 0.28 \times 10^{4}$	$6.04 \pm 0.68 \times 10^{-6}$

**Sample**	**Pd-ECM**	**$Pd_{0.99}Pt_{0.01}$-ECM**	**$Pd_{0.9}Pt_{0.1}$-ECM**	**$Pd_{0.75}Pt_{0.25}$-ECM**	**$Pd_{0.5}Pt_{0.5}$-ECM**
Conductivity ($S\ m^{-1}$)	$5.60 \pm 0.42 \times 10^{3}$	$4.69 \pm 0.21 \times 10^{3}$	$6.78 \pm 0.66 \times 10^{3}$	$8.91 \pm 0.06 \times 10^{3}$	$8.19 \pm 0.06 \times 10^{3}$

Table S2. Performance comparison of the existing Pt-based catalysts for hydrogen evolution reaction.

**Template**	**Material**	**Current density** @ –50 mV *vs*. RHE ($mA\ cm^{-2}_{geo}$)	**Mass activity** @ –50 mV *vs*. RHE ($A\ mg^{-1}_{Pt}$)	**Electrolyte**	**Ref**
VS_2 nanosheet	$1Pt/VS_2/CP$	-5^a	-0.7^a	0.5 M H_2SO_4	[S1]
TiO_2 nanosheet	$Pt\text{-}TiO_{2-x}$	-15^a	-0.2^a	0.5 M H_2SO_4	[S2]
N-doped carbon sphere	$Pt_1/NMHCS$	-15^a	–2.07	0.5 M H_2SO_4	[S3]
Carbon sphere	$Pt_{NP}/MHCS$	-6.5^a	–0.89		
Cu foam	Pt@Cu-0.3	-7^a	-	0.5 M H_2SO_4	[S4]
Octahedral carbon	PtCu-MoO_2@C	-30^a	–0.49	0.5 M H_2SO_4	[S5]
F-SnO_2 aerogel	F-SnO_2@Pt	-11.5^a	-9^a	0.5 M H_2SO_4	[S6]
Fibronectin (Biotemplate)	Au@Pt-ECM	–14.4	–0.52	0.5 M H_2SO_4	This work

a) When the actual value was not reported, we estimated the values from the figures.

References

[S1] J. Zhu, L. Cai, X. Yin, Z. Wang, L. Zhang, H. Ma, Y. Ke, Y. Du, S. Xi, A. T. S. Wee, Y. Chai, W. Zhang, *ACS Nano* **2020**, *14*, 5600.

[S2] K. M. Naik, E. Higuchi, H. Inoue, *Nanoscale* **2020**, *12*, 11055.

[S3] P. Kuang, Y. Wang, B. Zhu, F. Xia, C. W. Tung, J. Wu, H. M. Chen, J. Yu, *Adv. Mater.* **2021**, *33*, 2008599.

[S4] Y. Tan, R. Xie, S. Zhao, X. Lu, L. Liu, F. Zhao, C. Li, H. Jiang, G. Chai, D. J. L. Brett, P. R. Shearing, G. He, I. P. Parkin, *Adv. Funct. Mater.* **2021**, *31*, 2105579.

[S5] C. Zhang, P. Wang, W. Li, Z. Zhang, J. Zhu, Z. Pu, Y. Zhao, S. Mu, *J. Mater. Chem. A* **2020**, *8*, 19348.

[S6] T. Kim, S. B. Roy, S. Moon, S. H. Yoo, H. Choi, V. G. Parale, Y. Kim, J. Lee, S. C. Jun, K. Kang, S. H. Chun, K. Kanamori, H. H. Park, *ACS Nano* **2022**, *16*, 1625.

Table S3. Performance comparison of the existing Pd-based hydrogen sensing materials.

Material	Performance indicators				Empirically viable range of $[H_2]$ (%)	Ref
	$[H_2]$ (%)	Sensitivity (%)	Response time (s)	Recovery time (s)		
PdNi thin film	1.0	1.35	25	30	0.1 – 100	[S7]
Single Pd nanowires	2.4	7	< 30	< 30	0.0027 – 30	[S8]
PdNi thin film (MOTIFE)	2.0	3.92	0.67	-	0.08 – 10	[S9]
Pd nanotube array	1.0	3754	210	-	0.01 – 1	[S10]
Pd nanowires/ ZIF-8 (4 h)	1.0	3.47	7	10	0.1 – 1	[S11]
PdPt nanograins on nanofiber yarn	2.0	5	36	8	0.0001 – 4	[S12]
$Pd_{0.99}Pt_{0.01}$-ECM	1.0	2.68	31	75	0.007 – 1	This work

References

[S7] R. C. Hughes, W. K. Schubert, *J. Appl. Phys.* **1992**, *71*, 542.

[S8] P. Offermans, H. D. Tong, C. J. M. van Rijn, P. Merken, S. H. Brongersma, M. Crego-Calama, *Appl. Phys. Lett.* **2009**, *94*, 223110.

[S9] J. Lee, W. Shim, E. Lee, J.-S. Noh, W. Lee, *Angew. Chem. Int. Ed.* **2011**, *50*, 5301.

[S10] M. A. Lim, D. H. Kim, C.-O. Park, Y. W. Lee, S. W. Han, Z. Li, R. S. Williams, I. Park, *ACS Nano* **2012**, *6*, 590.

[S11] W.-T. Koo, S. Qiao, A. F. Ogata, G. Jha, J.-S. Jang, V. T. Chen, I.-D. Kim, R. M. Penner, *ACS Nano* **2017**, *11*, 9276.

[S12] D.-H. Kim, S.-J. Kim, H. Shin, W.-T. Koo, J.-S. Jang, J.-Y. Kang, Y. J. Jeong, I.-D. Kim, *ACS Nano* **2019**, *13*, 6071.

Table S4. The information about primary and secondary antibodies and their diluted concentrations. In the case of initial concentration could not be confirmed, only the dilution ratio was noted.

Product name	Vendor	Product number	Dilution ratio and Final concentration
Rabbit anti-Fibronectin antibody	Abcam	Ab2413	1:200
Rabbit anti-Collagen I antibody	Abcam	Ab21286	1:500
Rabbit anti-Laminin antibody	Abcam	Ab11575	1:500 to 1.4 μg/mL
Rabbit anti-Elastin antibody	Abcam	Ab21610	1:500
Rabbit anti-Tyrosine Hydroxylase antibody	Novus	NB300-109	1:500
Goat anti-Collagen IV antibody	Millipore	AB769	1:400 to 1 μg/mL
Chicken anti-MAP2 antibody	Abcam	Ab5392	1:500
Alexa Fluor 546- FluoroNanogold Fab' Goat anti-Rabbit antibody	Nanoprobes	7404	1:20 to 4 μg/mL
Alexa Fluor 647- FluoroNanogold IgG Fab' Rabbit anti-Goat antibody	Nanoprobes	7506	1:20 to 4 μg/mL
Alexa Fluor 488- 10 nm colloidal gold IgG Goat anti-Rabbit antibody	Thermo Fisher	A-31566	1:8 to 3.75 μg/mL
Alexa Fluor 546 Goat anti-Rabbit antibody	Thermo Fisher	A-11035	1:500 to 4 μg/mL
CF 633 Goat anti-Chicken antibody	Biotium	20126	1:500
DAPI	Sigma	D9542	1:1000 to 1 μg/mL

[1] E. M. Glebov, A. V. Kolomeets, I. P. Pozdnyakov, V. F. Plyusnin, V. P. Grivin, N. V. Tkachenko, H. Lemmetyinen, *RSC Adv.* **2012**, *2*, 5768.

[2] J. J. Cruywagen, R. J. Kriek, *J. Coord. Chem.* **2007**, *60*, 439.
